# Supplementary material for: Seeking and receiving help for mental health services among pregnant women in Ghana
Source: PLoS One. 2023 Mar 3;18(3):e0280496. doi: 10.1371/journal.pone.0280496 (PMC9983869; doi:10.1371/journal.pone.0280496)
Supplement: Attachment — Submitted filename: PONE-D-22-07154_R1.pdf [file pone.0280496.s003.pdf]

PLOS ONE

Seeking and Receiving Help for Mental Health Services Among Pregnant Women in Ghana

--Manuscript Draft--

**Manuscript Number:**	PONE-D-22-07154R1
**Article Type:**	Research Article
**Full Title:**	Seeking and Receiving Help for Mental Health Services Among Pregnant Women in Ghana
**Short Title:**	Mental Health Help seeking in pregnancy
**Corresponding Author:**	Samuel Adjorlolo<br>University of Ghana<br>Accra, GHANA
**Keywords:**	Perinatal Mental Health; Help-seeking; pregnant women; mental health disorders.
**Abstract:**	Objective : The heightened vulnerability of women to mental health issues during the period of pregnancy implies that seeking and receiving support for mental health services is a crucial factor in improving the emotional and mental well-being of pregnant women. The current study investigates the prevalence and correlates of seeking and receiving help for mental health services initiated by pregnant women and health professionals during pregnancy.<br>Design : Using a cross-sectional design and self-report questionnaires, data were collected from 702 pregnant women in the first, second and third trimesters from four health facilities in the Greater Accra region of Ghana. Data were analyzed using descriptive and inferential statistics.<br>Results : It was observed that 18.9% of pregnant women self-initiated help-seeking for mental health services whereas 64.8% reported that health professionals asked about their mental well-being, of which 67.7% were offered mental health support by health professionals. Diagnosis of medical conditions (i.e., hypertension and diabetes) in pregnancy, partner abuse, low social support, sleep difficulty and suicidal ideation significantly predicted the initiation of help-seeking for mental health services by pregnant women. Fear of vaginal delivery and COVID-19 concerns predicted the provision of mental health support to pregnant women by health professionals.<br>Conclusion : The low prevalence of individual-initiated help-seeking implies that health professionals have a high responsibility of supporting pregnant women achieve their mental health needs.
**Order of Authors:**	Samuel Adjorlolo
**Response to Reviewers:**	Seeking and Receiving Help for Mental Health Services Among Pregnant Women in Ghana<br>(PONE-D-22-07154)<br><br>Thank you for the reviewer comments. The response to the issues raised are highlighted in yellow ink for quick reference.<br><br>Reviewer #1's Comments:<br>Comment 1: The author has written on a very important subject titled "Seeking and Receiving Help for Mental Health Services Among Pregnant Women in Ghana". This in no doubt adds to the body of knowledge in the subject area. It will however be important for the author to address the following concerns:<br>Response: Thank you very much for the positive feedback.<br><br>Abstract<br>Introduction<br>Comment 2: Line 2 under the introduction-periods of pregnancy should be prenatal and not perinatal as perinatal includes the postpartum period as well.<br>Response: This has been corrected. Thank you.<br><br>Participants<br>Comment 3: What influenced the selection of the various proportions of the 702

Powered by Editorial Manager® and ProduXion Manager® from Aries Systems Corporation

pregnant women from the 4 different facilities indicated in the write-up.
Response: Across the facilities, the decision was to recruit as many participants as possible. The potential influence of facility-level factors on the study variables was recognized, and statistical plan was to control for this effect. Thus, a minimum of 50 participants per facility was envisaged for any meaningful statistical comparison.
Comment 4: What was the selection of the study facilities based on? The facilities selected were private, quasi government and CHAG hospitals. Do they not bias the patient population selected? Is this not one of the limitations of the study?
Response: Excellent observation. If you consider the study settings on the basis of ownership, it is apt to conclude on biasness. However, in terms of the demographics of participants who participated in the study, they are not entirely different from the general pool of pregnant women who attend government facilities. In fact, the health facilities serve pregnant from diverse socioeconomic background, an observation that mirror developments at government hospital. Nevertheless, we have included this as a potential limitation of the study as follows: The choice of the study settings does not include government owned health facilities and it is not clear whether similar findings would be obtained from government facilities.
Study design and data collection
Comment 5: The inclusion criteria of nulliparity/multiparity means that parity was not a deciding factor in selection and therefore this can be deleted.
Response: This has been deleted.
Comment 6: What sampling method was used to select the study participants?
Response: Convenience sampling was used to recruit participants who met the inclusion criteria. The manuscript has been revised to accommodate this as follows; Recruitment of the participant was based on convenience, availability, and willingness to participate in the study.

Predictor variables
Comment 7: What is the validity and reliability of using these 2 questions 1 I have been belittled by my partner (i.e., abuse) and (2) I experienced no attention from my partner as items for measuring intimate partner violence.
Response: These items were extracted from existing tool used to screen for IPV. These two items produced an internal consistency using Cronbach formula of 0.52. This low internal consistency is expected given the small number of items, hence limiting the amount of variations needed to high or acceptable internal consistency.

Data analysis and strategy
Comment 8: Last paragraph on page 12, the sentence will be clearer if framed as “Results were entered as Crude Odds ratios (COR) and adjusted Odds ratios (AOR).”
Response: This have been incorporated into the revised manuscript.

Results
Participants
Comment 9: In the first paragraph of this section, the author states that participants were recruited with pregnancy throughout all the trimesters. This is also stated in the abstract under methodology. It is not clear whether this selection was made with a plan at the beginning of the study and if this was the case, what was the exact plan to make sure that women were recruited from all the trimesters. Is it a case of what was realized after data was collected. This needs clarification.
Response: The plan for data collection was to include pregnant women across the trimesters. The focus was to investigate how mental health problems in the prenatal period relate to pregnancy outcome, explaining why information on participant folder numbers were obtained for a follow-up.

Comment 10: In table 2, under results, the author has a column captioned value. Please make this clear. Is it Chi square or p- square value?
Response: This has been corrected. The value represents chi-square value.

Comment 11: With the legends under table 3, the author indicates + = point-biserial correlation coefficients. This legend cannot be found in the table.
Response: In Table 3, + was attached to the continuous variables (e.g., social support, covid concern) to indicate that their correlations with the dichotomous variable was based on point-biserial correlations.

Powered by Editorial Manager® and ProduXion Manager® from Aries Systems Corporation

	Comment 12: In table 4, since the odds ratio and adjusted odds are both odds ratios, it is suggested that the bivariate odds is stated as Crude Odds ratio instead of just odds ratio.<br>Response: This has been incorporated into the revised manuscript.<br><br>Comment 13: The author states on page 15 under the predictors of mental health help seeking that "The statistical significance of the predictors did not change when demographic factors were controlled for." However if you closely examine table 5, there are several instances when statistical significance changed after accounting for other factors in the regression model. For instance under past medical history as a predictor for offered mental health support, the crude odds ratio was 2.42*** (1.511- 3.870) which was significant but the adjusted odds ratio was 1.21(0.608-2.412) which was not significant. There are several of these in table 5.<br>Response: Thank you for the excellent observation. This was an oversight. The statement has been deleted from the revised manuscript.<br><br>Discussion<br>Comment 14: In the discussion on page 17 and 18 under Health Professionals Involvement in Promoting Mental Health in Pregnancy, the author indicates that "The documented relationship between low education and risk for poor mental health (Ten Kate et al., 2017) could partly explain why pregnant women with secondary school education in this study reported that health professionals enquired about their emotional and mental wellbeing." Yet in the design of this study those with actual low level education, that is below secondary education level were left out due to language to English language as a barrier. The questionnaire could have been interpreted into other languages for them. The omission of that critical group is one of the key deficiencies of the study.<br>Response: Thank you for the observation. You are right about the omission of persons with below secondary education. Unfortunately, resource issues prevented their inclusion. Second, the literature seems to have focused extensively on pregnant women with poor education. Those with relatively high education (the so-called elite pregnant women) have not been granted enough research attention with respect to mental health. The study is contributing uniquely to this context. This was acknowledged as a limitation of the study.<br><br>Comment 15: On page 19, the author indicates that:" Efforts to persuade pregnant women capable of vaginal delivery to do so may involve offering them mental health support, including counselling and providing referral sources to allay fear and anxiety". Is the fear of vaginal delivery not better tackled as a comprehensive issue and not just a mental health issue? Issues to do with assurance of good monitoring during labour, issues to do with good pain relief etc.<br>Response: Your observation is correct. A comprehensive approach is certainly needed. However, given the context of the study, we can speak to mental health support since other support systems or packages were not explored.
**Additional Information:**	
**Question**	**Response**
**Financial Disclosure**<br><br>Enter a financial disclosure statement that describes the sources of funding for the work included in this submission. Review the submission guidelines for detailed requirements. View published research articles from *PLOS ONE* for specific examples.<br><br>This statement is required for submission and **will appear in the published article** if the submission is accepted. Please make	The author(s) received no specific funding for this work.

sure it is accurate.<br><br>**Unfunded studies**<br>Enter: *The author(s) received no specific funding for this work.*<br><br>**Funded studies**<br>Enter a statement with the following details:<br>• Initials of the authors who received each award<br>• Grant numbers awarded to each author<br>• The full name of each funder<br>• URL of each funder website<br>• Did the sponsors or funders play any role in the study design, data collection and analysis, decision to publish, or preparation of the manuscript?<br>• **NO** - Include this sentence at the end of your statement: *The funders had no role in study design, data collection and analysis, decision to publish, or preparation of the manuscript.*<br>• **YES** - Specify the role(s) played.<br><br>* typeset	
**Competing Interests**<br><br>Use the instructions below to enter a competing interest statement for this submission. On behalf of all authors, disclose any competing interests that could be perceived to bias this work—acknowledging all financial support and any other relevant financial or non-financial competing interests.<br><br>This statement is required for submission and **will appear in the published article** if the submission is accepted. Please make sure it is accurate and that any funding sources listed in your Funding Information later in the submission form are also declared in your Financial Disclosure statement.<br><br>View published research articles from *PLOS ONE* for specific examples.	The authors have declared that no competing interests exist.

Powered by Editorial Manager® and ProduXion Manager® from Aries Systems Corporation

**NO authors have competing interests**<br><br>Enter: *The authors have declared that no competing interests exist.*<br><br>**Authors with competing interests**<br><br>Enter competing interest details beginning with this statement:<br><br>*I have read the journal's policy and the authors of this manuscript have the following competing interests: [insert competing interests here]*<br><br>* typeset	
**Ethics Statement**<br><br>Enter an ethics statement for this submission. This statement is required if the study involved:<br><br>• Human participants<br>• Human specimens or tissue<br>• Vertebrate animals or cephalopods<br>• Vertebrate embryos or tissues<br>• Field research<br><br>Write "N/A" if the submission does not require an ethics statement.<br><br>General guidance is provided below. Consult the submission guidelines for detailed instructions. **Make sure that all information entered here is included in the Methods section of the manuscript.**	The study received ethics clearance from the Nugochi Memorial Institute for Medical Research, University of Ghana (NMIMR-IRB CPN 057/19-20).<br><br>Written informed consent was sought from the participants.

Powered by Editorial Manager® and ProduXion Manager® from Aries Systems Corporation

**Format for specific study types**<br><br>**Human Subject Research (involving human participants and/or tissue)**<br>• Give the name of the institutional review board or ethics committee that approved the study<br>• Include the approval number and/or a statement indicating approval of this research<br>• Indicate the form of consent obtained (written/oral) or the reason that consent was not obtained (e.g. the data were analyzed anonymously)<br><br>**Animal Research (involving vertebrate animals, embryos or tissues)**<br>• Provide the name of the Institutional Animal Care and Use Committee (IACUC) or other relevant ethics board that reviewed the study protocol, and indicate whether they approved this research or granted a formal waiver of ethical approval<br>• Include an approval number if one was obtained<br>• If the study involved *non-human primates*, add *additional details* about animal welfare and steps taken to ameliorate suffering<br>• If anesthesia, euthanasia, or any kind of animal sacrifice is part of the study, include briefly which substances and/or methods were applied<br><br>**Field Research**<br><br>Include the following details if this study involves the collection of plant, animal, or other materials from a natural setting:<br>• Field permit number<br>• Name of the institution or relevant body that granted permission	
**Data Availability**<br><br>Authors are required to make all data underlying the findings described fully available, without restriction, and from the time of publication. PLOS allows rare exceptions to address legal and ethical concerns. See the PLOS Data Policy and FAQ for detailed information.	Yes - all data are fully available without restriction

A Data Availability Statement describing where the data can be found is required at submission. Your answers to this question constitute the Data Availability Statement and **will be published in the article**, if accepted.<br><br>**Important:** Stating 'data available on request from the author' is not sufficient. If your data are only available upon request, select 'No' for the first question and explain your exceptional situation in the text box.<br><br>Do the authors confirm that all data underlying the findings described in their manuscript are fully available without restriction?	
**Describe where the data may be found in full sentences. If you are copying our sample text, replace any instances of XXX with the appropriate details.**<br><br>• If the data are **held or will be held in a public repository**, include URLs, accession numbers or DOIs. If this information will only be available after acceptance, indicate this by ticking the box below. For example: *All XXX files are available from the XXX database (accession number(s) XXX, XXX.*).<br>• If the data are all contained **within the manuscript and/or Supporting Information files**, enter the following: *All relevant data are within the manuscript and its Supporting Information files.*<br>• If neither of these applies but you are able to provide **details of access elsewhere**, with or without limitations, please do so. For example:<br><br>*Data cannot be shared publicly because of [XXX]. Data are available from the XXX Institutional Data Access / Ethics Committee (contact via XXX) for researchers who meet the criteria for access to confidential data.*<br><br>*The data underlying the results presented in the study are available from (include the name of the third party*	The data underlying the results presented in the study are available from DOI: 10.17632/bmgbndy38g.2

and contact information or URL).<br>• This text is appropriate if the data are owned by a third party and authors do not have permission to share the data.<br><br>* typeset	
Additional data availability information:	

Powered by Editorial Manager® and ProduXion Manager® from Aries Systems Corporation

Manuscript Click here to access/download;Manuscript;Manuscript.docx

Seeking and Receiving Help for Mental Health Services among Pregnant Women in Ghana

Samuel Adjorlolo, PhD

Department of Mental Health Nursing, College of Health Sciences, University of Ghana, P. O. Box LG 43, Legon Accra.

Corresponding Author

Samuel Adjorlolo; Department of Mental Health Nursing, College of Health Sciences, University of Ghana, P. O. Box LG 43, Legon Accra. Email: sadjorlolo@ug.edu.gh, Tel: +233204197158.

Seeking and Receiving Help for Mental Health Services among Pregnant Women in Ghana

Abstract

Objective: The heightened vulnerability of women to mental health issues during the period of pregnancy implies that seeking and receiving support for mental health services is a crucial factor in improving the emotional and mental well-being of pregnant women. The current study investigates the prevalence and correlates of seeking and receiving help for mental health services initiated by pregnant women and health professionals during pregnancy.

Design: Using a cross-sectional design and self-report questionnaires, data were collected from 702 pregnant women in the first, second and third trimesters from four health facilities in the Greater Accra region of Ghana. Data were analyzed using descriptive and inferential statistics.

Results: It was observed that 18.9% of pregnant women self-initiated help-seeking for mental health services whereas 64.8% reported that health professionals asked about their mental well-being, of which 67.7% were offered mental health support by health professionals. Diagnosis of medical conditions (i.e., hypertension and diabetes) in pregnancy, partner abuse, low social support, sleep difficulty and suicidal ideation significantly predicted the initiation of help-seeking for mental health services by pregnant women. Fear of vaginal delivery and COVID-19 concerns predicted the provision of mental health support to pregnant women by health professionals.

Conclusion: The low prevalence of individual-initiated help-seeking implies that health professionals have a high responsibility of supporting pregnant women achieve their mental health needs.

Keywords: Perinatal mental health; help-seeking; pregnant women; mental health disorders.

Introduction

There is ample literature regarding the burden of mental health problems during the periods of pregnancy (i.e., prenatal period) and after delivery (postnatal period); a burden that is highly elevated in low- and middle-income countries mainly due to the preponderance of risk factors such as poverty, food insecurity, pollution, migration, conflict, climate change, and gender-based violence [1, 2]. Depression, for instance, is estimated to affect about 11.3% of pregnant women and 18.9% of mothers after birth in Africa, whereas the prevalence of pre- and postnatal anxiety has been estimated at 14.8% and 14%, respectively [3]. Perinatal mental illness has been associated with adverse pregnancy outcomes such as preterm birth, intrauterine growth restriction [4] and childhood behavioral and emotional problems [5, 6]. These adverse pregnancy outcomes have been implicated in maternal and child mortality in Ghana [7].

While the provision of mental health support is central to improving the mental well-being of pregnant women [8, 9], this is also contingent on help-seeking for mental health services. Rickwood, Deane (10) conceptualized help-seeking as a social transaction between intrapersonal and interpersonal processes. The intrapersonal process is concerned with an individual's level of awareness and demonstration of the need to seek help. This process is premised on the assumption that an individual has the capacity and competency to differentiate among different mental states and seek help when they perceive or experience mental health issues. The awareness of this intrapersonal need is expected to lead to a *self-initiated help-seeking* for mental health services. Under the self-initiated help-seeking framework, pregnant women are expected to activate the mental healthcare pathway by initiating contact with and seeking help from (mental) health professionals to manage their emotional problems. Self-

initiated mental health help-seeking largely attests to the willingness of a pregnant woman to share her emotional problems with others but also the desire to seek and receive help.

However, research has shown that pregnant women are less likely to seek support for mental health on their own for reasons such as fear of being shamed, embarrassed, socially isolated and stigmatized [11-14]. Other barriers include lack of information about services and access to care, lack of knowledge regarding maternal mental health and preference for informal sources of support [14]. The stigmatization of mental health and psychiatric facilities in Ghana, for instance, is among the incentives pushing individuals to seek mental health support from informal sources, including religious leaders and traditional healers[15]. Therefore, health professionals have important roles in ameliorating the emotional and mental well-being of pregnant women [8, 9]. Indeed, evidence-based clinical and practice guidelines directs health professionals to prioritize and promote the mental health of pregnant women. The United Kingdom National Institute for Health Care Excellence (NICE) guidelines categorically places the responsibility of maternal mental health in the hands of health professionals. These professionals are expected to initiate conversations around pregnancy and mental health, to enquire about the emotional well-being of pregnant women as well as offer mental health support, including referral services [16]. The American college of Obstetricians and Gynecologists (ACOG) patient safety bundle on maternal mental health has similarly tasked the health delivery system and professionals to proactively take steps to improve the wellbeing of pregnant women. Some recommended measures include establishing a response protocol and identifying screening tools, gathering data via administering valid screening tools and activating the necessary response system for mental health support. Such interactions can lead to a reduction in the stigma associated with perinatal mental health, serving as an avenue to address

other common barriers such as lack of knowledge, attitudes and beliefs that affect maternal mental health [14]. Research has shown that pregnant women are more likely to seek help for their mental health issues when health professionals take steps to foster trust, enquire about perinatal mental health issues, and offer the needed support [12, 17].

Although perinatal mental health has gained traction in the literature [14, 18], no study has systematically explored pregnant women self-initiating help-seeking for mental health services as well as health professionals' involvement in maternal mental health simultaneously. Previous studies have centered on help-seeking among pregnant women diagnosed with or experiencing mental health challenges [13, 19, 20]. There is a general lack of evidence on help-seeking among pregnant women in general. Extending the discussions and empirical literature to all pregnant women, rather than focusing on those diagnosed with mental disorders, is consistent with the call for a population approach to promoting mental health [21, 22] and the recommendation by the UK's NICE and ACOG patient safety bundle on maternal mental health. This approach also has the opportunity to increase mental health literacy in pregnancy and empower pregnant women to proactively safeguard their mental well-being through initiatives such as reducing maternal stressors and seeking help for emotional discomfort. Together, these can have a considerable impact on the health and well-being of their children and the next generations, given the relationship between maternal mental well-being and infant, child and adolescent development and psychopathology [5, 6, 21].

In addition to the limited studies focusing on self-initiated mental health help-seeking among pregnant women [13, 19, 20], the involvement of health professionals in promoting maternal mental health by, first and foremost, enquiring about pregnant women's emotional well-being and, secondly, providing mental health support, including referral services, needs to

be examined. Although recent studies have explored challenges affecting the delivering of perinatal mental health services by health professionals [8, 9], it is not clear the extent to which these professionals are supporting pregnant women in meeting their emotional well-being needs. Moreover, the range of factors that is influencing self-initiated mental health help-seeking among pregnant women and health professionals' involvement in promoting maternal mental health is unclear. If known, they could form the basis for intervention programming.

Lastly, there is limited empirical evidence on seeking and receiving help for mental health services in pregnancy among women in low- and middle income countries, including Ghana, despite the reported high burden of mental illness [23]. Management of mental health issues in Ghana mostly involve a mixture of psychiatric and faith-based and traditional healing services[15]. Evidence suggests that faith-based and traditional healing services are mostly the first point of contact because of a confluence of factors Pathways to mental health treatment in Ghana: challenging biomedical methods from herbal-and faith-healing perspectives[15]. These include the tendency to ascribe spiritual meanings to mental health issues and the restriction of psychiatric services to the three mental health hospitals that are not evenly distributed across the 16 regions in Ghana[24, 25]. Efforts to decentralize and integrate the provision of mental health services into primary healthcare is underway across regional and district hospitals across the country. Owing to their supposed complementarity, discussions have been advanced for a working collaboration between biomedical, faith and traditional healing pathways for the management of mental disorders[15]. Thus, pregnant women, like other patients, could invoke psychiatric-hospital treatment pathways or the faith-based and traditional healing services. The Ghanaian health professionals, like their counterparts in high-income countries, have a responsibility of ensuring and responding to the emotional and well-being issues of their

clients/patients. In addition to providing basic mental health services such as screening and counseling, nurses and midwives have the responsibility to refer clients/patients suspected of mental disorders to mental health professionals for support and care. Seeking help for and receiving mental health support from health professionals would help to improve the mental well-being of pregnant women. However, as noted previously, there is limited data on pregnant women seeking help on their own as well as receiving mental health support from health professionals in Ghana and other LMICs.

Research addressing these topical issues is needed to provide evidence base to develop a holistic strategy to ensure that pregnant women with mental health challenges have the appropriate services and support systems required. Consequently, the overarching goal of the study is to investigate mental health help-seeking among pregnant women by addressing the aforementioned research gaps. The study objectives were: (1) to investigate the number of pregnant women who initiated mental health help-seeking on their own, who were asked about their mental well-being and/or were offered mental health support by health professionals and (2) to determine the influence of factors culled from medical history, intimate partner violence, COVID-19 worries, social support, fear of delivery, sleep difficulty and suicidal ideation domains on self-initiated help-seeking and health professional involvement in mental health support

Methods

Ethical approval

The study received ethics clearance from the Noguchi Memorial Institute for Medical Research, University of Ghana (NMIMR-IRB CPN 057/19-20).

Participants

The data were collected as part of a study investigating maternal mental health in Ghana. Data were gathered from 702 pregnant women recruited from the antenatal clinics of four health facilities, namely University of Ghana hospital (*n* = 175, 24.9%), Alpha hospital (*n* = 239, 34%), Sanford Clinic (*n* = 87, 12.4%) and St-Gregory Catholic hospital (*n* = 201, 28.6%) in the Greater Accra region of Ghana. These facilities, located across different geographical locations within the Greater Accra region, are patronized by pregnant women of different demographic backgrounds.

Study design and data collection

A cross-sectional design was used. Participants were recruited from the health facilities based on their availability and willingness to participate in the study. The inclusion criteria for participating in the study were age ≥18 years, and completion of senior high school diploma or equivalent (i.e., 12 years of formal education). This requirement assured the participants were proficient in English. It was most practical to use English questionnaires as there are more than 10 languages spoken in the Greater Accra region of Ghana. The exclusion criteria were diagnosis of mental disorder during pregnancy and history of mental health disorder. Data were collected by research assistants (RAs) who received training in salient issues relating to research with pregnant women, including privacy, confidentiality, non-judgmental attitude, recruitment, and questionnaire administration. At each facility, nurses or midwives were identified as facility-based focal persons. These individuals supported the recruitment process by introducing the research assistants to the participants attending the antenatal clinics. Thereafter, the RAs approached and discussed the study with individual pregnant woman. Participants were recruited on based on convenience. The participants, who often congregate at the antenatal clinics of the

facilities, were informed about the purpose and duration of the study, their responsibilities for participating in the study, ethical issues such as confidentiality, consent, anonymity and benefits of participation. Questions raised by the participants were responded to by the research team to allay fears, anxieties and encourage participation in the study.

Prior to completing the questionnaire, the participants read and signed the consent form, together with the RAs. The "broad consent" gave the research team the permission to obtain data on pregnancy outcomes and other pertinent obstetric information after delivery from the participants' folders, where necessary. The folder/hospital identity numbers of the participants were recorded to facilitate subsequent matching of information. The questionnaires were completed individually and independently. The RAs were present to provide the needed support to the participants. Once completed, the questionnaires were handed over to the RAs. Data collection lasted for approximately eight weeks, from September to October 2020. The COVID-19 precautionary measures such as wearing of nose masks and use of alcohol-based hand sanitizers were strictly adhered to.

Data Collection Measures

Outcome variables: The outcome variables for the study were self-initiated help-seeking and health professional involvement in maternal mental. Self-initiated help-seeking was measured with a single item: *I have sought help for emotional or mental health issues while pregnant in the last 3 months*. Based on the recommendations from the NICE (2014) and ACOG (2016) guidelines, the participants responded to two items to estimate the extent to which health professionals were involved in promoting their mental well-being: (1) *Nurses and midwives have asked me about my mental health or emotional well-being* and (2) *Nurses or midwives have offered me support such as counselling and referral for my mental health or emotional well-*

being needs. The response options for self-initiated help-Seeking and health professional involvement were as follows: Never; Yes, once; Yes, twice and Yes, several times. These were subsequently recoded into two categories for each item: “Never” (coded 0) and “at least once” (coded 1).

Predictor Variables

A total of seven variables were considered as predictor variables in this study. These were described below.

Obstetric History: The participants were asked whether they have been diagnosed with medical conditions such as hypertension and diabetes in their previous pregnancy and current pregnancy. Responses were dichotomous: Yes or No.

Fear of Delivery: This was measured with two items: 1) Fear of vaginal delivery and (2) fear of caesarean section. The participants responded by choosing either “Yes” or “No”.

Intimate Partner Violence: This was operationalized using two items from the literature [26, 27]: (1) I have been belittled by my partner (i.e., abuse) and (2) I experienced no attention from my partner (i.e., neglect). Responses were dichotomous: Yes or No.

COVID-19 Concerns: The literature has revealed heightened worries and fears about the coronavirus in the general population [28]. Among pregnant women, this fear could be extended to the impact of contracting the coronavirus on the pregnancy and baby. We therefore assessed COVID-19 concerns using two items that were scored on a four-point Likert response scale from Not all (0) to Very often (3). The first item relates to whether the participants were worried about contracting the virus and the second involved whether the participants were worried that their babies could develop some birth or developmental abnormalities should they contract the coronavirus. A total score was obtained by summing the responses, with higher scores indicating

more COVID-19 concerns. A Cronbach's Alpha of 0.92 was obtained for the two-item "COVID-19 concern" scale.

Sleep Difficulty: This was measured by asking about (1) difficulty to fall asleep while in bed and (2) difficulty to stay asleep through the night. The items were extracted from the existing literature [29] and were scored using a four-point Likert response format ranging from Not at all (0) to Very often (3). Responses to each item were added to create a total score, with higher scores indicating more sleep difficulty. The Cronbach's Alpha for the sleep difficulty scale was 0.86.

Suicidal Ideation: Following a review of the literature [30], three items were extracted to index suicidal ideation as follows: Have you (1) ever thought that life wasn't worth living; (2) ever thought about killing yourself; and (3) ever attempted to kill yourself? The response options ranged from Never (0) to Yes, several times (3). Total scores obtained by summing the responses on the scale ranged from 0 to 9, with higher scores reflecting more suicidal ideation. The Cronbach's Alpha for the suicidal ideation scale was 0.71.

Social Support: This was also measured with three items culled from the existing literature [26] as follows: (1) Someone is around when I am in need; (2) I have someone with whom I can share my joy and sorrow; and (3) I have received emotional support from my partner. The participants responded to the items using a four-point Likert scale, from Strongly disagree (1) to Strongly agree (4). The responses were added together with total score ranging from 3 to12. Higher scores indicated more social support. A Cronbach's Alpha of 0.87 was recorded for the Social Support Scale.

Data analysis strategy: Data were analyzed using SPSS Version 23 (IBM.corp), with a two-tailed statistical significance set at 0.05. The outcome variables were analyzed using

descriptive statistics, namely frequency and percentages. Chi-square was used to investigate the bivariate relationship between the outcome variables and categorical predictor and demographic variables. The relationship between outcome and continuous predictor variables (e.g., age, social support, COVID-19 worries, sleep difficulty and suicidal ideation) were examined using point-biserial correlation. The point-biserial correlation is used to examine the relationship between a continuous predictor variable and a dichotomous outcome variables. The coefficient for point-biserial correlation ranges from -1 to 1 and is interpreted in the same way as Pearson or Spearman correlation coefficients. Lastly, binary logistic regression was used to examine the predictors of the outcome variables. In the first of the series of logistic regression analysis, the regression model contained only one predictor. This was followed by the model of individual predictors controlling for demographic variables. Lastly, all the predictor variables were entered into the model concurrently, controlling for demographic variables. Results were reported as crude odds ratios (COR) and adjusted odds ratios (AOR) with 95% confidence intervals (95% CI). Only the variables that demonstrated significant bivariate correlations with the outcome variables were included in the binomial logistic regression.

Results

Participants

The participants were recruited from the various pregnancy periods (1st trimester = 63, 9%; 2nd trimester = 315, 44.9% and 3rd trimester = 324, 46.2%). Out of the total participants recruited, 125 (18%) and 569 (82%) were single and married, respectively. More than half (n = 358, 53%) completed senior high school/equivalent (at least 12 years of education), 168 (24.9%) completed post-secondary school (additional 2 or 3 years of schooling from senior high school) whereas 22.1% (n = 149) completed university education. In terms of employment, 48.6% (n =

322) reportedly worked in government or private establishments (so-called formal employment), 29.6 (*n* = 196) in informal employment and 21.8% (*n* = 144) were unemployed. The average age of the participants was 30 years (*SD* = 5.54).

Intimate partner violence appeared highly prevalent, with 36.4% (*n* = 686) and 86% (*n* = 686) of the study participants reported partner abuse and neglect, respectively. A total of 34.2% (*n* = 634) and 66.8% (*n* = 633) participants were fearful of vaginal delivery and delivery by caesarean section, respectively. Data on obstetric history showed that 12.7% (*n* = 693) of the participants were diagnosed with medical conditions, namely hypertension and diabetes while pregnant in the past, whereas14.5% (*n* = 691) were diagnosed with these conditions in the present pregnancy.

Prevalence of Mental Health Help-Seeking

It was observed that 18.9% of the participants initiated mental health help-seeking on their own, suggesting majority (*n* = 557, 81.1%) did not seek mental health support in the last three months prior to the study. In contrast, more than half (*n* = 444, 64.8%) of the participants reported that nurses and midwives enquired about their mental and emotional well-being at least once. In terms of support, 47.1% (*n* = 320) of the participants reported that they were offered mental health support by the nurses and midwives. A statistically significant correlation was observed between being asked about mental wellbeing and being offered mental health support ($\chi^2 = 209.11$, $p < .001$). About 67.7% of the participants who were asked about their mental wellbeing were offered some mental health support.

[**INSERT TABLE 1 HERE**]

When analyzed across the various demographic factors, varying results were obtained (Table 2). For instance, self-initiated mental health help-seeking did not differ significantly across the trimesters of pregnancy. However, participants in the second trimester were more likely to have been asked about their mental well-being (z = 2.2, $p < 0.01$) and/or received some mental health support from health professionals (z = 3.0, $p < 0.001$). Health professionals were also more likely to ask about mental well-being or provide mental health support to participants who completed only senior high school.

[INSERT TABLE 2 HERE]

Intercorrelations among Study Variables

Table 3 contained a summary of the results of the intercorrelations among the study variables. As can be seen, the study variables were significantly correlated. The outcome variables correlated with several of the predictor variables. Self-initiated mental health help-seeking correlated significantly with past medical history ($\chi^2 = 34.48$, p < .01), present medical history ($\chi^2 = 27.97$, p < .01), partner neglect ($\chi^2 = 11.24$, p < .01), partner abuse ($\chi^2 = 26.32$, p < .01), social support ($r = -.36$, p < .01), sleep difficulty ($r = .21$, p < .01) and suicidal ideation ($r = .31$, p < .01). Health professionals were more likely to ask about mental well-being and provide mental health support to participants diagnosed with obstetric history, those who feared vaginal delivery, were victims of partner abuse, had difficulties sleeping and harbored suicidal ideations.

[INSERT TABLE 3 HERE]

Predictors of Mental Health Help-Seeking

Different sets of binary logistic regressions were performed for each of the outcome variables. The results of the model containing the individual predictors and model of individual predictors controlling for demographic factors are shown in Table 4. The majority of the

variables individually and independently predicted the outcome variables. For example, participants who experienced partner abuse were 2.73 ($\chi^2 = 25.19$, $p < 0.001$), 1.61 ($\chi^2 = 7.61$, $p < 0.01$) and 1.56 ($\chi^2 = 7.64$, $p < .01$) more likely to seek mental health support on their own, to have been asked about their mental well-being and to have been offered mental health support by health professionals, respectively.

[INSERT TABLE 4 HERE]

When all the predictors were entered into the model simultaneously while controlling for demographic variables, the results, summarized in Table 5, showed that the history of diagnosis of medical condition in a previous pregnancy (AOR = 3.80, $p < 0.01$), partner abuse (AOR = 1.88, $p < .05$), social support (AOR = 0.80, p < 0.001), sleep difficulty (AOR = 1.35, $p < .01$) and suicidal ideation (AOR = 1.22, $p < 0.01$) emerged as the significant predictors of self-initiated mental health help-seeking. None of the predictor variables significantly predicted the likelihood that participants were asked about their mental well-being by health professionals. In contrast, fear of vaginal delivery (AOR = 1.91, $p < 0.05$) and COVID-19 concerns (AOR = 1.19, $p < .05$) were significantly associated with health professionals offering mental health support to the participants.

[INSERT TABLE 5 HERE]

Discussion

Mental health challenges are common among pregnant and postpartum women across the globe. However, studies evaluating mental health help-seeking behaviors for this vulnerable population are limited globally and virtually non-existent in Ghana and other low- and middle-income countries.

Self-initiated mental health help-seeking in pregnancy

Since the period of pregnancy is associated with physiological changes and the likelihood of exposure to stressful factors such as financial difficulties and occupational hazards that have been implicated in perinatal mental health [27, 31, 32], changes in emotional and mental well-being are expected. It is postulated that pregnant women who initiate a mental health help-seeking process by themselves are more likely to have the ability not only to detect changes in their emotional state and mental well-being but more importantly to act on these changes by seeking help and keeping to a treatment schedule. In this study, only a small number of pregnant women reportedly sought help on their own in the last three month prior to the study. This finding is consistent with an earlier finding [11]. In addition to the social (e.g., stigma and embarrassment), instrumental (e.g., financial restraints) and structural barriers (e.g., lack of information) affecting help-seeking for mental health services by pregnant women [14], the limited access to mental health services in Ghana [24] could contribute to the low help-seeking for mental health services among pregnant women. The provision of mental health services is concentrated at the three public psychiatric institutions in the country, two of which are located in the Greater Accra region of Ghana. Owing to stigma, most people are not willing to visit these facilities for support. Although there are ongoing efforts to decentralize and integrate the provision of mental health services into primary healthcare [33], the limited publicity of these services means that several people, including pregnant women, may not be aware of their availability. The traditional world view of Ghanaians regarding demons or supernatural forces as causes of mental disorders places additional constraints on pregnant women's willingness to initiate mental health help-seeking from friends and family members and health professionals, despite the documented benefits of doing so [13].

The study showed that few pregnant women who reportedly sought help on their own for mental health problems were more likely to be women with obstetric history, were victims of intimate partner violence, had sleep difficulty, experienced suicidal ideation and reported low social support. This observation largely supports the existing literature on the relationship between exposure to risk factors and utilization of mental health services [27, 31, 32].

Health Professionals Involvement in Promoting Mental Health in Pregnancy

The observation that pregnant women infrequently seek mental health support on their own volition as reported in this and previous studies [11] calls for more pragmatic efforts from health professionals. In this study, more than half of the pregnant women reported that nurses/midwives asked about their emotional or mental well-being. This observation is largely consistent with an earlier study in Ghana in which more than half of the nurses and midwives reported that they were involved in promoting maternal mental health such as asking pregnant women about their emotional well-being [9]. Demographically, pregnant women in the second trimester and those with a high school diploma or equivalent were more likely to be asked about their mental and emotional well-being. Anecdotally, most pregnant women in Ghana begin attending antenatal clinics in their second trimester. This raises the possibility that a comprehensive intake assessment undertaken for first time antenatal clinic attendees may comprise an assessment of their mental and emotional well-being. The documented relationship between low education and risk for poor mental health [34] could partly explain why pregnant women with secondary school education in this study reported that health professionals enquired about their emotional and mental well-being.

When demographic variables were controlled for, sleep difficulty emerged as the robust predictor of the pregnant women being asked about mental and emotional well-being as well as

being offered mental health support such as counselling and referral services. Poor sleep not only negatively impacts mental health [35], but it is also a symptom of poor mental health (Anderson & Bradley, 2013). Therefore, sleep difficulty is likely to come to the attention of health professionals as a contributory factor to the mental health challenges reported by pregnant women. Pregnant women who feared vaginal delivery and expressed concerns about COVID-19 were reportedly offered mental health support. In a population-based cohort study, fear of vaginal delivery was strongly associated with a preference for elective caesarean section [36]. Estimated to range from 7%-27%, elective caesarean section is a major concern among researchers, policy-makers, stakeholders and health professionals owing to the associated surgical complications [37] and perhaps costs and consequences for future pregnancies. Efforts to persuade pregnant women capable of vaginal delivery to do so may involve offering them mental health support, including counselling and providing referral sources to allay fear and anxiety. A similar argument could be advanced for concerns regarding COVID-19. That is, health professionals may offer mental health support to reduce the fears and anxiety surrounding COVID-19 as a way of encouraging the continuous use of antenatal services and to promote delivery at health facilities in the COVID-19 pandemic.

Lastly, nurses and midwives enquired about the mental health or emotional wellbeing of more than half of the participants. In terms of support, about 67% of the participants received some mental health support. Perhaps, those who received support were those who indicated poor mental health or emotional distress to the nurses and midwives. There is also the possibility that some pregnant women may not be offered mental health support, although they may indicate problems with their mental health or emotional well-being. While the reasons accounting for this were not explored in this study, a previous study identified barriers hampering the involvement

of nurses and midwives in perinatal mental health in Ghana. These include lack of knowledge about mental health in pregnancy, unavailability of mental health services, and lack of a clear mental healthcare pathway [8].

Limitations

The study findings should be evaluated in light of the following limitations. The cross-sectional design adopted in this study does not permit commentaries on causal relationship. The recruitment of participants with at least senior high school education for the study implies that the findings may not be applicable to those with less formal education. Therefore, the application of the study findings should be restricted to pregnant women who have attended at least secondary education in Ghana. As noted previously, most pregnant women start antenatal clinics in their second trimester, making it difficult to obtain a representation of this category of women. The findings could have been different with equal or similar number of pregnant women across the three trimesters. The use of convenience sampling increases the risk of selection bias that may impact the study result negatively. Indeed, because the participants were self-selecting, there is a possibility that only those with unique characteristics (e.g., interest in research) agreed to participate in the study. This line of reasoning further limits the application of the study findings to a broader population of pregnant women with at least senior secondary education. The choice of the study settings does not include government owned health facilities and it is not clear whether similar findings would be obtained from government facilities. Lastly, although single or few-item measures are capable of representing complex variables, multiple-item questionnaires are known to have superior psychometric properties, which could have influenced the findings reported here [38, 39].

Conclusion

Pregnancy is a period of vulnerability for women to experience poor mental health. Several pregnancy-related stressors are unavoidable and many that are preventable continue to exist and manifest in diverse forms. Providing mental health support early enough to pregnant women could help to mitigate the impact of stressors on their mental and emotional well-being as well as the development of their children. While it is important to promote self-initiated mental health help-seeking, it is equally imperative to involve health professionals in the provision of mental health support, as advocated by the WHO's task shifting concept [40]. Both individual and health professional-initiated activation of the mental health pathway will ultimately benefit pregnant women and their unborn babies. With evidence suggesting high mental health illiteracy among pregnant women [41], greater responsibility is placed on health professionals to intervene in providing mental health services to pregnant women, as advocated for by the NICE service management and clinical guidance for antenatal and postnatal mental health [16]. Health professionals should be well positioned to identify or detect pregnant women in need of mental health services and activate the pathway towards seeking help. The findings reported here re-emphasize the need for profiling pregnant women. To understand pregnant women who are likely to seek mental health support on their own, the study suggests that attention should be paid to those with the following background: History of hypertension or diabetes in the past and present pregnancies, victims of intimate partner violence, sleep difficulty, suicidal ideation and low social support. Health professionals can target pregnant women with the aforementioned experiences, in addition to those who feared vaginal delivery and expressed concern about COVID-19, to improve their mental and emotional well-being.

Acknowledgement

The project is an OPERA (Optimal Pregnancy Environment Risk Association) project. We acknowledge the Canadian Queen Elizabeth II Diamond Jubilee Scholarships-Advanced Scholars (QES-AS) program. The QES is managed through a unique partnership of Universities Canada, the Rideau Hall Foundation (RHF), Community Foundations of Canada (CFC) and Canadian universities. The QES-AS is made possible with financial support from IDRC and SSHRC."

Declaration of interests

The authors declare that they have no known competing financial interests or personal relationships that could have appeared to influence the work reported in this paper.

References

1. Grose RG, Roof KA, Semenza DC, Leroux X, Yount KM. Mental health, empowerment, and violence against young women in lower-income countries: A review of reviews. Aggression and violent behavior. 2019;46:25-36.

2. Cohen E, Ware LJ, Prioreschi A, Draper C, Bosire E, Lye SJ, et al. Material and Relational Difficulties: The Impact of the Household Environment on the Emotional Well-Being of Young Black Women Living in Soweto, South Africa. Journal of Family Issues. 2020;41(8):1307-32.

3. Sawyer A, Ayers S, Smith H. Pre-and postnatal psychological wellbeing in Africa: a systematic review. Journal of affective disorders. 2010;123(1-3):17-29.

4. Ghimire U, Papabathini SS, Kawuki J, Obore N, Musa TH. Depression during pregnancy and the risk of low birth weight, preterm birth and intrauterine growth restriction-an updated meta-analysis. Early Human Development. 2020;152:105243.

5. Rees S, Channon S, Waters CS. The impact of maternal prenatal and postnatal anxiety on children's emotional problems: a systematic review. European Child & Adolescent Psychiatry. 2019;28(2):257-80. doi: 10.1007/s00787-018-1173-5.

6. Slomian J, Honvo G, Emonts P, Reginster J-Y, Bruyère O. Consequences of maternal postpartum depression: A systematic review of maternal and infant outcomes. Women's Health. 2019;15:1745506519844044.

7. Weobong B, ten Asbroek AH, Soremekun S, Gram L, Amenga-Etego S, Danso S, et al. Association between probable postnatal depression and increased infant mortality and morbidity: findings from the DON population-based cohort study in rural Ghana. BMJ open. 2015;5(8).

8. Adjorlolo S, Aziato L. Barriers to addressing mental health issues in childbearing women in Ghana. Nursing Open. 2020;7(6):1779-86.

9. Adjorlolo S, Aziato L, Akorli VV. Promoting maternal mental health in Ghana: An examination of the involvement and professional development needs of nurses and midwives. Nurse education in practice. 2019;39:105-10.

10. Rickwood D, Deane FP, Wilson CJ, Ciarrochi J. Young people's help-seeking for mental health problems. Australian e-journal for the Advancement of Mental health. 2005;4(3):218-51.

11. Redshaw M, Henderson J. Who is actually asked about their mental health in pregnancy and the postnatal period? Findings from a national survey. BMC psychiatry. 2016;16(1):322.

12. Jesse DE, Dolbier CL, Blanchard A. Barriers to seeking help and treatment suggestions for prenatal depressive symptoms: Focus groups with rural low-income women. Issues in mental health nursing. 2008;29(1):3-19.

13. Barrera AZ, Nichols AD. Depression help-seeking attitudes and behaviors among an Internet-based sample of Spanish-speaking perinatal women. Revista panamericana de salud publica. 2015;37:148-53.

14. Jones A. Help seeking in the perinatal period: A review of barriers and facilitators. Social work in public health. 2019;34(7):596-605.

15. Badu E, Mitchell R, O'Brien AP. Pathways to mental health treatment in Ghana: Challenging biomedical methods from herbal-and faith-healing perspectives. International Journal of Social Psychiatry. 2019;65(6):527-38.

16. NICE. Antenatal and Postnatal Mental Health: Clinic Management and Service Guidance. London: 2014.

17. Reilly N, Harris S, Loxton D, Chojenta C, Forder P, Austin M-P. The impact of routine assessment of past or current mental health on help-seeking in the perinatal period. Women and Birth. 2014;27(4):e20-e7.

18. Smith MS, Lawrence V, Sadler E, Easter A. Barriers to accessing mental health services for women with perinatal mental illness: systematic review and meta-synthesis of qualitative studies in the UK. BMJ open. 2019;9(1):e024803.

19. Holt C, Milgrom J, Gemmill AW. Improving help-seeking for postnatal depression and anxiety: a cluster randomised controlled trial of motivational interviewing. Archives of women's mental health. 2017;20(6):791-801.

20. Da Costa D, Zelkowitz P, Nguyen T-V, Deville-Stoetzel J-B. Mental health help-seeking patterns and perceived barriers for care among nulliparous pregnant women. Archives of women's mental health. 2018;21(6):757-64.

21. Purtle J, Nelson KL, Counts NZ, Yudell M. Population-Based approaches to mental health: history, strategies, and evidence. Annual Review of Public Health. 2020;41:201-21.

22. Fuhrer R, Keyes KM. Population Mental Health in the 21st Century: Time to Act. American Public Health Association; 2019.

23. Whiteford HA, Degenhardt L, Rehm J, Baxter AJ, Ferrari AJ, Erskine HE, et al. Global burden of disease attributable to mental and substance use disorders: findings from the Global Burden of Disease Study 2010. The lancet. 2013;382(9904):1575-86.

24. Adjorlolo S, Abdul-Nasiru I, Chan HC, Bambi LE. Mental health professionals' attitudes toward offenders with mental illness (insanity acquittees) in Ghana. International journal of offender therapy and comparative criminology. 2018;62(3):629-54.

25. Ibrahim A, Hor S, Bahar OS, Dwomoh D, McKay MM, Esena RK, et al. Pathways to psychiatric care for mental disorders: a retrospective study of patients seeking mental health services at a public psychiatric facility in Ghana. International journal of mental health systems. 2016;10(1):1-11.

26. Molarius A, Berglund K, Eriksson C, Eriksson HG, Lindén-Boström M, Nordström E, et al. Mental health symptoms in relation to socio-economic conditions and lifestyle factors–a population-based study in Sweden. BMC public health. 2009;9(1):1-9.

27. Mandal SK, Hooker L, Vally H, Taft A. Partner violence and postnatal mental health: cross-sectional analysis of factors associated with depression and anxiety in new mothers. Australian journal of primary health. 2018;24(5):434-40.

28. Mertens G, Gerritsen L, Duijndam S, Salemink E, Engelhard IM. Fear of the coronavirus (COVID-19): Predictors in an online study conducted in March 2020. Journal of Anxiety Disorders. 2020;74:102258.

29. Kurtis MM, Balestrino R, Rodriguez-Blazquez C, Forjaz MJ, Martinez-Martin P. A review of scales to evaluate sleep disturbances in movement disorders. Frontiers in neurology. 2018;9:369.

30. Innamorati M, Pompili M, Serafini G, Lester D, Erbuto D, Amore M, et al. Psychometric properties of the suicidal history self-rating screening scale. Archives of Suicide Research. 2011;15(1):87-92.

31. Howard LM, Oram S, Galley H, Trevillion K, Feder G. Domestic violence and perinatal mental disorders: a systematic review and meta-analysis. PLoS Med. 2013;10(5):e1001452.

32. Leonardsson M, San Sebastian M. Prevalence and predictors of help-seeking for women exposed to spousal violence in India–a cross-sectional study. BMC women's health. 2017;17(1):99.

33. Akerele O. The decentralization of the ghanaian mental health system through the non-profit sector: a case to improve access to care and disrupt the “othering” of the mentally ill? Intersect: The Stanford Journal of Science, Technology, and Society. 2020;13(3).

34. Ten Kate J, De Koster W, Van der Waal J. Why are depressive symptoms more prevalent among the less educated? The relevance of low cultural capital and cultural entitlement. Sociological Spectrum. 2017;37(2):63-76.

35. Dixon CL. Sleep Deprivation & Pregnancy Related Risks. International Journal of Childbirth Education. 2014;29(3).

36. Størksen HT, Garthus-Niegel S, Adams SS, Vangen S, Eberhard-Gran M. Fear of childbirth and elective caesarean section: a population-based study. BMC pregnancy and childbirth. 2015;15(1):1-10.

37. Betrán AP, Ye J, Moller A-B, Zhang J, Gülmezoglu AM, Torloni MR. The increasing trend in caesarean section rates: global, regional and national estimates: 1990-2014. PloS one. 2016;11(2):e0148343.

38. Konstabel K, Lönnqvist J-E, Leikas S, García Velázquez R, Qin H, Verkasalo M, et al. Measuring single constructs by single items: Constructing an even shorter version of the “Short Five” personality inventory. PloS one. 2017;12(8):e0182714.

39. Cook CL, Perri III M. Single-item vs multiple-item measures of stage of change in compliance with prescribed medications. Psychological reports. 2004;94(1):115-24.

40. WHO. Task Shifting: Global Recommendations and Guidelines. Geneva: 2008.

41. Spedding MF, Stein DJ, Naledi T, Sorsdahl K. Pregnant women's mental health literacy and perceptions of perinatal mental disorders in the Western Cape, South Africa. Mental Health & Prevention. 2018;11:16-23.

Table 1: Prevalence of Self and Health Professional-Initiated Help-seeking for Mental Health among Pregnant Women (n = 702).

Variables/Response	Never	Yes	Once	Twice	Several times
	n (%)	*n* (%)	*n* (%)	*n* (%)	*n* (%)
**Self-Initiated Help-Seeking**					
Mental health seeking in last 3 months **(*n* = 687)**	557(81.1)	130(18.9)	83(12.1)	30(4.4)	17(2.5)
**Health Professional-Initiated Help-Seeking**					
Asked about mental health issues **(*n* = 685)**	241(35.2)	444(64.8)	227(33.1)	101(14.7)	116(16.9)
Offered mental health support **(*n*= 679)**	359(52.9)	320(47.1)	160(23.6)	78(11.5)	82(12.1)

Table 2: Frequencies and Chi Square Results for Demographic Correlates of the Outcome Variables.

Source	Self-Initiated Mental Health Help Seeking			Health Professional Initiated Mental Health Help Seeking			Health Professional Providing Mental Health Support		
	No *n* (%)	Yes *n* (%)	Value	No *n* (%)	Yes *n* (%)	Value	No *n* (%)	Yes *n* (%)	Value
**Age+**	-	-	.01, ns	-	-	.03, ns	-	-	.14**
**Research Setting**			96.07***			87.76***			208.11***
Legon	144 (21)	28(4.1)		75(10.9)	93(13.6)		122(18.0)	45(6.6)	
Alpha	210(37.7)	23(17.7)		122(17.8)	110(16.1)		174(25.6)	55(8.1)	
Sanford	37(5.4)	49(7.1)		18(2.6)	68(9.9)		38(5.6)	46(6.8)	
St-Gregory	166(24.2)	30(4.4)		26(3.8)	173(25.3)		25(3.7)	174(25.6)	
Total	557(81.1)	130(18.9)		241(35.2)	444(64.8)		359(52.9)	320(47.1)	
**Trimester**			.39, ns			23.96***			31.70***
First	52(7.6)	11(1.6)		25(3.6)	36(5.3)		36(5.3)	25(3.7)	
Second	245(35.7)	61(8.9)		77(11.2)	228(33.3)		124(18.3)	179(26.4)	
Third	260(35.8)	58(8.4)		139(20.3)	180(26.3)		199(29.3)	116(17.1)	
Total	557(81.1)	130(18.9)		241(35.2)	444(64.8)		359(52.9)	320(47.1)	
**Education**			4.40, ns			46.65***			48.80***
SHS/below	276(41.6)	72(10.9)		102(15.5)	249(37.7)		154(23.5)	194(29.7)	
Diploma or equivalence	133(20.1)	33(5)		43(6.5)	121(18.3)		79(12.1)	84(12.8)	
First degree & above	129(19.5)	19(2.9)		85(12.9)	60(9.1)		112(17.1)	31(4.7)	
Total	538(81.3)	124(18.7)		230(34.8)	430(65.2)		345(52.8)	309(47.2)	
**Marital Status**			16.37***			3.02, ns			1.84, ns
Single	86(12.6)	39(5.7)		35(5.2)	89(13.1)		58(8.6)	65(9.7)	
Married	469(68.9)	87(12.8)		202(29.8)	352(51.9)		296(44)	253(37.6)	
Total	555(81.5)	126(18.5)		237(35)	441(65)		354(52.7)	318(47.3)	
**Employment**			2.91, ns			.44, ns			8.22*
Formal	251(38.4)	69(10.6)		113(17.4)	201(30.9)		179(27.8)	134(20.8)	
Informal	157(24)	33(5.1)		65(10)	129(19.8)		85(13.2)	107(16.6)	
Unemployed	121(18.5)	22(3.4)		52(8)	90(13.8)		76(11.8)	63(9.8)	
Total	529(81)	124(19)		230(35.4)	420(64.6)		340(52.8)	304(47.2)	

* $p < .05$; ** $p < .01$, *** $p < .01$, ns = not significant.

Table 3: Chi Square and Point-Biserial Correlation Interrelations Results for Study Variables

	1	2	3	4	5	6	7	8	9	10	11	12
**Self-Initiated Help-Seeking**												
**1.** Last 3 month	1											
**Professional Initiated Help-Seeking**												
**2.** Asked	27.72**	1										
**3.** Support offered	18.31**	209.11**	1									
**Medical History**												
**4.** Past	34.48**	5.39*	14.16**	1								
**5.** Present	27.97**	10.07**	13.26**	320.79**	1							
**Fear of Delivery**												
**6.** Vaginal delivery	27.08**	16.98**	50.28**	1.03	.63	1						
**7.** Cesarean Section	2.01	8.60**	26.04**	2.76	.01	28.24**	1					
**Intimate Partner Violence**												
**8.** Partner Neglect	11.24**	.46	.51	11.34**	13.40**	2.48	.30	1				
**9.** Partner abuse	26.32**	7.67**	7.67**	11.30**	15.13**	3.90*	17.39	.40	1			
**10. Social Support+**	-.36**	-.08*	-.03	.14**	.21**	.14**	.05	.25**	-.31**	1		
**11. COVID-19+ concern**	.09*	.26***	.41**	-.21***	-.13**	-.26**	-.33**	-.04	.17**	.06	1	
**12. Sleep Difficulty+**	.21**	.22***	.31**	-.10**	-.12**	-.30**	-.24**	-.11**	.14**	-.08*	.47**	1
**13. Suicidal Ideation+**	.31**	.11**	.08*	-.09*	-.12**	-.17**	-.07	-.21**	.16**	-.39**	.05	.17**

* p < .05; ** p < .01; + = point-biserial correlation coefficients.

Table 4: Predictors of Mental Health Help-Seeking

	Self- Help-seeking		Asked about Mental health		Offered Mental Health Support	
	OR (95% CI)	AOR (95% CI)	OR (95% CI)	AOR (95% CI)	OR (95% CI)	AOR (95% CI)
**Medical history**						
Past	3.90***(2.419-6.289)	3.58***(2.071-6.184)	1.83*(1.093-3.079)	1.18(0.639-2.158)	2.42***(1.511-3.870)	1.21(0.608-2.412)
Present	3.13**(1.970-4.979)	2.26**(1.328-3.847)	2.25**(1.349-3.74)	1.67(0.922-3.021)	2.24***(1.439-3.474)	1.40(0.736-2.669)
**Fear of delivery**						
Vaginal delivery	2.91***(1.926-4.395)	1.94**(1.186-3.185)	2.17***(1.496-3.157)	1.13(0.714-1.788)	3.47***(2.444-4.938)	2.13**(1.254-3.603)
C- section	1.38(0.883-2.156)	1.29(0.770-2.153)	1.68**(1.185-2.371)	1.01(0.672-1.504)	2.45***(1.728-3.463)	1.17(0.721-1.912)
**Partner violence**						
Partner neglect	.44**(0.274-720)	.47*(0.266-838)	1.17(0.746-1.829)	1.38(0.807-2.351)	.85(0.553-1.319)	.87(0.461-1.655)
Partner abuse	2.73***(1.845-4.045)	2.12*(1.362-3.301)	1.61**(1.148-2.260)	1.12(0.753-1.667)	1.56**(1.139-2.145)	1.11(0.697-1.750)
**Social support**	.70***(0.648-.762)	.77***(0.695-845)	.93*(0.869-.996)	.95(0.867-1.034)	.98(0.920-1.043)	.97(0.869-1.071)
**COVID-19**	1.10*(1.014-1.202)	1.15*(1.017-1.035)	1.30***(1.205-1.411)	1.09(.987-1.211)	1.51***(1.391-1.631)	1.21**(1.082-1.359)
**Sleep difficulty**	1.38***(1.227-1.548)	1.37***(1.191-1.586)	1.36***(1.221-1.515)	1.15*(1.011-1.305)	1.52***(1.364-1.686)	1.18*(1.020-1.363)
**Suicidal ideation**	1.61***(1.415-1.839)	1.40***(1.208-1.619)	1.21(1.056-1.378)	1.16(0.997-1.349)	1.13*(1.006-1.261)	1.12(0.954-1.303)

* $p < 0.05$; ** $p < 0.01$, *** $p < 0.001$; C –section = Caesarean section

Adjusted odds ratios were obtained after controlling for demographic variables that evinced significant effect on the outcome variables (Table 2).

Table 5: Predictors of Mental Health Help-Seeking, Controlling for Individual Predictors and Demographic Variables.

	Help-seeking last 1 month	Asked about Mental health	Offered Mental Health Support
	AOR (95% CI)	AOR (95% CI)	AOR (95% CI)
**Medical history**			
Past	3.80** (1.636-8.842)	.74(.320-1.706)	.82(.317-2.127)
Present	0.75 (0.326-1.747)	1.97(.858-4.509)	1.24(.496-3.088)
**Fear of delivery**			
Vaginal delivery	1.59(.903-2.792)	1.07(.662-1.743)	1.91*(1.097-3.312)
Caesarean section	.78(.422-1.433)	.82(.526-1.275)	1.06(.630-1.768)
**Partner violence**			
Partner neglect	.74(.367-1.471)	1.46(.775-2.757)	.85(.392-1.823)
Partner abuse	1.88*(1.091-3.288)	1.11(.703-1.750)	1.11(.655-1.882)
**Social support**	.80***(.711-908)	.96(.863-1.063)	.95(.842-1.080)
**COVID-19 worries**	1.07(.905-1.261)	1.09(.971-1.234)	1.19*(1.038-1.354)
**Sleep difficulty**	1.35**(1.125-1.626)	1.04(.896-1.201)	1.05(.881-1.239)
**Suicidal ideation**	1.22**(1.092-1.449)	1.13(.950-1.339)	1.09(.916-1.307)

* p < .05; ** p < .01, *** p < .001

Demographic variables that evinced significant effect on the outcome variables (Table 2) were controlled for.

Revised Manuscript with Track Changes

Seeking and Receiving Help for Mental Health Services among Pregnant Women in Ghana

Samuel Adjorlolo, PhD

Department of Mental Health Nursing, College of Health Sciences, University of Ghana, P. O. Box LG 43, Legon Accra.

Corresponding Author

Samuel Adjorlolo; Department of Mental Health Nursing, College of Health Sciences, University of Ghana, P. O. Box LG 43, Legon Accra. Email: sadjorlolo@ug.edu.gh, Tel: +233204197158.

Seeking and Receiving Help for Mental Health Services among Pregnant Women in Ghana

Abstract

Objective: The heightened vulnerability of women to mental health issues during the period of pregnancy implies that seeking and receiving support for mental health services is a crucial factor in improving the emotional and mental well-being of pregnant women. The current study investigates the prevalence and correlates of seeking and receiving help for mental health services initiated by pregnant women and health professionals during pregnancy.

Design: Using a cross-sectional design and self-report questionnaires, data were collected from 702 pregnant women in the first, second and third trimesters from four health facilities in the Greater Accra region of Ghana. Data were analyzed using descriptive and inferential statistics.

Results: It was observed that 18.9% of pregnant women self-initiated help-seeking for mental health services whereas 64.8% reported that health professionals asked about their mental well-being, of which 67.7% were offered mental health support by health professionals. Diagnosis of medical conditions (i.e., hypertension and diabetes) in pregnancy, partner abuse, low social support, sleep difficulty and suicidal ideation significantly predicted the initiation of help-seeking for mental health services by pregnant women. Fear of vaginal delivery and COVID-19 concerns predicted the provision of mental health support to pregnant women by health professionals.

Conclusion: The low prevalence of individual-initiated help-seeking implies that health professionals have a high responsibility of supporting pregnant women achieve their mental health needs.

Keywords: Perinatal mental health; help-seeking; pregnant women; mental health disorders.

Introduction

There is ample literature regarding the burden of mental health problems during the periods of pregnancy (i.e., prenatal period) and after delivery (postnatal period); a burden that is highly elevated in low- and middle-income countries mainly due to the preponderance of risk factors such as poverty, food insecurity, pollution, migration, conflict, climate change, and gender-based violence [1, 2]. Depression, for instance, is estimated to affect about 11.3% of pregnant women and 18.9% of mothers after birth in Africa, whereas the prevalence of pre- and postnatal anxiety has been estimated at 14.8% and 14%, respectively [3]. Perinatal mental illness has been associated with adverse pregnancy outcomes such as preterm birth, intrauterine growth restriction [4] and childhood behavioral and emotional problems [5, 6]. These adverse pregnancy outcomes have been implicated in maternal and child mortality in Ghana [7].

While the provision of mental health support is central to improving the mental well-being of pregnant women [8, 9], this is also contingent on help-seeking for mental health services. Rickwood, Deane (10) conceptualized help-seeking as a social transaction between intrapersonal and interpersonal processes. The intrapersonal process is concerned with an individual's level of awareness and demonstration of the need to seek help. This process is premised on the assumption that an individual has the capacity and competency to differentiate among different mental states and seek help when they perceive or experience mental health issues. The awareness of this intrapersonal need is expected to lead to a *self-initiated help-seeking* for mental health services. Under the self-initiated help-seeking framework, pregnant women are expected to activate the mental healthcare pathway by initiating contact with and seeking help from (mental) health professionals to manage their emotional problems. Self-

initiated mental health help-seeking largely attests to the willingness of a pregnant woman to share her emotional problems with others but also the desire to seek and receive help.

However, research has shown that pregnant women are less likely to seek support for mental health on their own for reasons such as fear of being shamed, embarrassed, socially isolated and stigmatized [11-14]. Other barriers include lack of information about services and access to care, lack of knowledge regarding maternal mental health and preference for informal sources of support [14]. The stigmatization of mental health and psychiatric facilities in Ghana, for instance, is among the incentives pushing individuals to seek mental health support from informal sources, including religious leaders and traditional healers[15]. Therefore, health professionals have important roles in ameliorating the emotional and mental well-being of pregnant women [8, 9]. Indeed, evidence-based clinical and practice guidelines directs health professionals to prioritize and promote the me

ntal health of pregnant women. The United Kingdom National Institute for Health Care Excellence (NICE) guidelines categorically places the responsibility of maternal mental health in the hands of health professionals. These professionals are expected to initiate conversations around pregnancy and mental health, to enquire about the emotional well-being of pregnant women as well as offer mental health support, including referral services [16]. The American college of Obstetricians and Gynecologists (ACOG) patient safety bundle on maternal mental health has similarly tasked the health delivery system and professionals to proactively take steps to improve the wellbeing of pregnant women. Some recommended measures include establishing a response protocol and identifying screening tools, gathering data via administering valid screening tools and activating the necessary response system for mental health support (ACOG, 2016). Such interactions can lead to a reduction in the stigma associated with perinatal mental

health, serving as an avenue to address other common barriers such as lack of knowledge, attitudes and beliefs that affect maternal mental health [14]. Research has shown that pregnant women are more likely to seek help for their mental health issues when health professionals take steps to foster trust, enquire about perinatal mental health issues, and offer the needed support [12, 17].

Although perinatal mental health has gained traction in the literature [14, 18], no study has systematically explored pregnant women self-initiating help-seeking for mental health services as well as health professionals' involvement in maternal mental health simultaneously. Previous studies have centered on help-seeking among pregnant women diagnosed with or experiencing mental health challenges [13, 19, 20]. There is a general lack of evidence on help-seeking among pregnant women in general. Extending the discussions and empirical literature to all pregnant women, rather than focusing on those diagnosed with mental disorders, is consistent with the call for a population approach to promoting mental health [21, 22] and the recommendation by the UK's NICE and ACOG patient safety bundle on maternal mental health. This approach also has the opportunity to increase mental health literacy in pregnancy and empower pregnant women to proactively safeguard their mental well-being through initiatives such as reducing maternal stressors and seeking help for emotional discomfort. Together, these can have a considerable impact on the health and well-being of their children and the next generations, given the relationship between maternal mental well-being and infant, child and adolescent development and psychopathology [5, 6, 21].

In addition to the limited studies focusing on self-initiated mental health help-seeking among pregnant women [13, 19, 20], the involvement of health professionals in promoting maternal mental health by, first and foremost, enquiring about pregnant women's emotional

well-being and, secondly, providing mental health support, including referral services, needs to be examined. Although recent studies have explored challenges affecting the delivering of perinatal mental health services by health professionals [8, 9], it is not clear the extent to which these professionals are supporting pregnant women in meeting their emotional well-being needs. Moreover, the range of factors that is influencing self-initiated mental health help-seeking among pregnant women and health professionals' involvement in promoting maternal mental health is unclear. If known, they could form the basis for intervention programming.

Lastly, there is limited empirical evidence on seeking and receiving help for mental health services in pregnancy among women in low- and middle income countries, including Ghana, despite the reported high burden of mental illness [23]. Management of mental health issues in Ghana mostly involve a mixture of psychiatric and faith-based and traditional healing services[15]. Evidence suggests that faith-based and traditional healing services are mostly the first point of contact because of a confluence of factors Pathways to mental health treatment in Ghana: challenging biomedical methods from herbal-and faith-healing perspectives[15]. These include the tendency to ascribe spiritual meanings to mental health issues and the restriction of psychiatric services to the three mental health hospitals that are not evenly distributed across the 16 regions in Ghana[24, 25]. Efforts to decentralize and integrate the provision of mental health services into primary healthcare is underway across regional and district hospitals across the country. Owing to their supposed complementarity, discussions have been advanced for a working collaboration between biomedical, faith and traditional healing pathways for the management of mental disorders[15]. Thus, pregnant women, like other patients, could invoke psychiatric-hospital treatment pathways or the faith-based and traditional healing services. The Ghanaian health professionals, like their counterparts in high-income countries, have a

responsibility of ensuring and responding to the emotional and well-being issues of their clients/patients. In addition to providing basic mental health services such as screening and counseling, nurses and midwives have the responsibility to refer clients/patients suspected of mental disorders to mental health professionals for support and care. Seeking help for and receiving mental health support from health professionals would help to improve the mental well-being of pregnant women. However, as noted previously, there is limited data on pregnant women seeking help on their own as well as receiving mental health support from health professionals in Ghana and other LMICs.

Research addressing these topical issues is needed to provide evidence base to develop a holistic strategy to ensure that pregnant women with mental health challenges have the appropriate services and support systems required. Consequently, the overarching goal of the study is to investigate mental health help-seeking among pregnant women by addressing the aforementioned research gaps. The study objectives were: (1) to investigate the number of pregnant women who initiated mental health help-seeking on their own, who were asked about their mental well-being and/or were offered mental health support by health professionals and (2) to determine the influence of factors culled from medical history, intimate partner violence, COVID-19 worries, social support, fear of delivery, sleep difficulty and suicidal ideation domains on self-initiated help-seeking and health professional involvement in mental health support

Methods

Ethical approval

The study received ethics clearance from the Noguchi Memorial Institute for Medical Research, University of Ghana (NMIMR-IRB CPN 057/19-20).

Participants

The data were collected as part of a study investigating maternal mental health in Ghana. Data were gathered from 702 pregnant women recruited from the antenatal clinics of four health facilities, namely University of Ghana hospital (n = 175, 24.9%), Alpha hospital (n = 239, 34%), Sanford Clinic (n = 87, 12.4%) and St-Gregory Catholic hospital (n = 201, 28.6%) in the Greater Accra region of Ghana. These facilities, located across different geographical locations within the Greater Accra region, are patronized by pregnant women of different demographic backgrounds.

Study design and data collection

A cross-sectional design was used. Participants were recruited from the health facilities based on their availability and willingness to participate in the study. The inclusion criteria for participating in the study were age ≥18 years, and completion of senior high school diploma or equivalent (i.e., 12 years of formal education). This requirement assured the participants were proficient in English. It was most practical to use English questionnaires as there are more than 10 languages spoken in the Greater Accra region of Ghana. The exclusion criteria were diagnosis of mental disorder during pregnancy and history of mental health disorder. Data were collected by research assistants (RAs) who received training in salient issues relating to research with pregnant women, including privacy, confidentiality, non-judgmental attitude, recruitment, and questionnaire administration. At each facility, nurses or midwives were identified as facility-based focal persons. These individuals supported the recruitment process by introducing the research assistants to the participants attending the antenatal clinics. Thereafter, the RAs

approached and discussed the study with individual pregnant woman. Participants were recruited on based on convenience. The participants, who often congregate at the antenatal clinics of the facilities, were informed about the purpose and duration of the study, their responsibilities for participating in the study, ethical issues such as confidentiality, consent, anonymity and benefits of participation. Questions raised by the participants were responded to by the research team to allay fears, anxieties and encourage participation in the study.

Prior to completing the questionnaire, the participants read and signed the consent form, together with the RAs. The "broad consent" gave the research team the permission to obtain data on pregnancy outcomes and other pertinent obstetric information after delivery from the participants' folders, where necessary. The folder/hospital identity numbers of the participants were recorded to facilitate subsequent matching of information. The questionnaires were completed individually and independently. The RAs were present to provide the needed support to the participants. Once completed, the questionnaires were handed over to the RAs. Data collection lasted for approximately eight weeks, from September to October 2020. The COVID-19 precautionary measures such as wearing of nose masks and use of alcohol-based hand sanitizers were strictly adhered to.

Data Collection Measures

Outcome variables: The outcome variables for the study were self-initiated help-seeking and health professional involvement in maternal mental. Self-initiated help-seeking was measured with a single item: *I have sought help for emotional or mental health issues while pregnant in the last 3 months*. Based on the recommendations from the NICE (2014) and ACOG (2016) guidelines, the participants responded to two items to estimate the extent to which health professionals were involved in promoting their mental well-being: (1) *Nurses and midwives have*

asked me about my mental health or emotional well-being and (2) *Nurses or midwives have offered me support such as counselling and referral for my mental health or emotional well-being needs*. The response options for self-initiated help-Seeking and health professional involvement were as follows: Never; Yes, once; Yes, twice and Yes, several times. These were subsequently recoded into two categories for each item: “Never” (coded 0) and “at least once” (coded 1).

Predictor Variables

A total of seven variables were considered as predictor variables in this study. These were described below.

Obstetric History: The participants were asked whether they have been diagnosed with medical conditions such as hypertension and diabetes in their previous pregnancy and current pregnancy. Responses were dichotomous: Yes or No.

Fear of Delivery: This was measured with two items: 1) Fear of vaginal delivery and (2) fear of caesarean section. The participants responded by choosing either “Yes” or “No”.

Intimate Partner Violence: This was operationalized using two items from the literature [26, 27]: (1) I have been belittled by my partner (i.e., abuse) and (2) I experienced no attention from my partner (i.e., neglect). Responses were dichotomous: Yes or No.

COVID-19 Concerns: The literature has revealed heightened worries and fears about the coronavirus in the general population [28]. Among pregnant women, this fear could be extended to the impact of contracting the coronavirus on the pregnancy and baby. We therefore assessed COVID-19 concerns using two items that were scored on a four-point Likert response scale from Not all (0) to Very often (3). The first item relates to whether the participants were worried about contracting the virus and the second involved whether the participants were worried that their

babies could develop some birth or developmental abnormalities should they contract the coronavirus. A total score was obtained by summing the responses, with higher scores indicating more COVID-19 concerns. A Cronbach's Alpha of 0.92 was obtained for the two-item "COVID-19 concern" scale.

Sleep Difficulty: This was measured by asking about (1) difficulty to fall asleep while in bed and (2) difficulty to stay asleep through the night. The items were extracted from the existing literature [29] and were scored using a four-point Likert response format ranging from Not at all (0) to Very often (3). Responses to each item were added to create a total score, with higher scores indicating more sleep difficulty. The Cronbach's Alpha for the sleep difficulty scale was 0.86.

Suicidal Ideation: Following a review of the literature [30], three items were extracted to index suicidal ideation as follows: Have you (1) ever thought that life wasn't worth living; (2) ever thought about killing yourself; and (3) ever attempted to kill yourself? The response options ranged from Never (0) to Yes, several times (3). Total scores obtained by summing the responses on the scale ranged from 0 to 9, with higher scores reflecting more suicidal ideation. The Cronbach's Alpha for the suicidal ideation scale was 0.71.

Social Support: This was also measured with three items culled from the existing literature [26] as follows: (1) Someone is around when I am in need; (2) I have someone with whom I can share my joy and sorrow; and (3) I have received emotional support from my partner. The participants responded to the items using a four-point Likert scale, from Strongly disagree (1) to Strongly agree (4). The responses were added together with total score ranging from 3 to12. Higher scores indicated more social support. A Cronbach's Alpha of 0.87 was recorded for the Social Support Scale.

Data analysis strategy: Data were analyzed using SPSS Version 23 (IBM.corp), with a two-tailed statistical significance set at 0.05. The outcome variables were analyzed using descriptive statistics, namely frequency and percentages. Chi-square was used to investigate the bivariate relationship between the outcome variables and categorical predictor and demographic variables. The relationship between outcome and continuous predictor variables (e.g., age, social support, COVID-19 worries, sleep difficulty and suicidal ideation) were examined using point-biserial correlation. The point-biserial correlation is used to examine the relationship between a continuous predictor variable and a dichotomous outcome variables. The coefficient for point-biserial correlation ranges from -1 to 1 and is interpreted in the same way as Pearson or Spearman correlation coefficients. Lastly, binary logistic regression was used to examine the predictors of the outcome variables. In the first of the series of logistic regression analysis, the regression model contained only one predictor. This was followed by the model of individual predictors controlling for demographic variables. Lastly, all the predictor variables were entered into the model concurrently, controlling for demographic variables. Results were reported as crude odds ratios (COR) and adjusted odds ratios (AOR) with 95% confidence intervals (95% CI). Only the variables that demonstrated significant bivariate correlations with the outcome variables were included in the binomial logistic regression.

Results

Participants

The participants were recruited from the various pregnancy periods (1st trimester = 63, 9%; 2nd trimester = 315, 44.9% and 3rd trimester = 324, 46.2%). Out of the total participants recruited, 125 (18%) and 569 (82%) were single and married, respectively. More than half (*n* = 358, 53%) completed senior high school/equivalent (at least 12 years of education), 168 (24.9%)

completed post-secondary school (additional 2 or 3 years of schooling from senior high school) whereas 22.1% (*n* = 149) completed university education. In terms of employment, 48.6% (*n* = 322) reportedly worked in government or private establishments (so-called formal employment), 29.6 (*n* = 196) in informal employment and 21.8% (*n* = 144) were unemployed. The average age of the participants was 30 years (*SD* = 5.54).

Intimate partner violence appeared highly prevalent, with 36.4% (*n* = 686) and 86% (*n* = 686) of the study participants reported partner abuse and neglect, respectively. A total of 34.2% (*n* = 634) and 66.8% (*n* = 633) participants were fearful of vaginal delivery and delivery by caesarean section, respectively. Data on obstetric history showed that 12.7% (*n* = 693) of the participants were diagnosed with medical conditions, namely hypertension and diabetes while pregnant in the past, whereas14.5% (*n* = 691) were diagnosed with these conditions in the present pregnancy.

Prevalence of Mental Health Help-Seeking

It was observed that 18.9% of the participants initiated mental health help-seeking on their own, suggesting majority (*n* = 557, 81.1%) did not seek mental health support in the last three months prior to the study. In contrast, more than half (*n* = 444, 64.8%) of the participants reported that nurses and midwives enquired about their mental and emotional well-being at least once. In terms of support, 47.1% (*n* = 320) of the participants reported that they were offered mental health support by the nurses and midwives. A statistically significant correlation was observed between being asked about mental wellbeing and being offered mental health support ($\chi^2 = 209.11$, $p < .001$). About 67.7% of the participants who were asked about their mental wellbeing were offered some mental health support.

[**INSERT TABLE 1 HERE**]

When analyzed across the various demographic factors, varying results were obtained (Table 2). For instance, self-initiated mental health help-seeking did not differ significantly across the trimesters of pregnancy. However, participants in the second trimester were more likely to have been asked about their mental well-being (z = 2.2, $p < 0.01$) and/or received some mental health support from health professionals (z = 3.0, $p < 0.001$). Health professionals were also more likely to ask about mental well-being or provide mental health support to participants who completed only senior high school.

[INSERT TABLE 2 HERE]

Intercorrelations among Study Variables

Table 3 contained a summary of the results of the intercorrelations among the study variables. As can be seen, the study variables were significantly correlated. The outcome variables correlated with several of the predictor variables. Self-initiated mental health help-seeking correlated significantly with past medical history ($\chi^2 = 34.48$, p < .01), present medical history ($\chi^2 = 27.97$, p < .01), partner neglect ($\chi^2 = 11.24$, p < .01), partner abuse ($\chi^2 = 26.32$, p < .01), social support ($r = -.36$, p < .01), sleep difficulty ($r = .21$, p < .01) and suicidal ideation ($r = .31$, p < .01). Health professionals were more likely to ask about mental well-being and provide mental health support to participants diagnosed with obstetric history, those who feared vaginal delivery, were victims of partner abuse, had difficulties sleeping and harbored suicidal ideations.

[INSERT TABLE 3 HERE]

Predictors of Mental Health Help-Seeking

Different sets of binary logistic regressions were performed for each of the outcome variables. The results of the model containing the individual predictors and model of individual

predictors controlling for demographic factors are shown in Table 4. The majority of the variables individually and independently predicted the outcome variables. For example, participants who experienced partner abuse were 2.73 ($\chi^2 = 25.19$, $p < 0.001$), 1.61 ($\chi^2 = 7.61$, $p < 0.01$) and 1.56 ($\chi^2 = 7.64$, $p < .01$) more likely to seek mental health support on their own, to have been asked about their mental well-being and to have been offered mental health support by health professionals, respectively.

[INSERT TABLE 4 HERE]

When all the predictors were entered into the model simultaneously while controlling for demographic variables, the results, summarized in Table 5, showed that the history of diagnosis of medical condition in a previous pregnancy (AOR = 3.80, $p < 0.01$), partner abuse (AOR = 1.88, $p < .05$), social support (AOR = 0.80, p < 0.001), sleep difficulty (AOR = 1.35, $p < .01$) and suicidal ideation (AOR = 1.22, $p < 0.01$) emerged as the significant predictors of self-initiated mental health help-seeking. None of the predictor variables significantly predicted the likelihood that participants were asked about their mental well-being by health professionals. In contrast, fear of vaginal delivery (AOR = 1.91, $p < 0.05$) and COVID-19 concerns (AOR = 1.19, $p < .05$) were significantly associated with health professionals offering mental health support to the participants.

[INSERT TABLE 5 HERE]

Discussion

Mental health challenges are common among pregnant and postpartum women across the globe. However, studies evaluating mental health help-seeking behaviors for this vulnerable population are limited globally and virtually non-existent in Ghana and other low- and middle-income countries.

Self-initiated mental health help-seeking in pregnancy

Since the period of pregnancy is associated with physiological changes and the likelihood of exposure to stressful factors such as financial difficulties and occupational hazards that have been implicated in perinatal mental health [27, 31, 32], changes in emotional and mental well-being are expected. It is postulated that pregnant women who initiate a mental health help-seeking process by themselves are more likely to have the ability not only to detect changes in their emotional state and mental well-being but more importantly to act on these changes by seeking help and keeping to a treatment schedule. In this study, only a small number of pregnant women reportedly sought help on their own in the last three month prior to the study. This finding is consistent with an earlier finding [11]. In addition to the social (e.g., stigma and embarrassment), instrumental (e.g., financial restraints) and structural barriers (e.g., lack of information) affecting help-seeking for mental health services by pregnant women [14], the limited access to mental health services in Ghana [24] could contribute to the low help-seeking for mental health services among pregnant women. The provision of mental health services is concentrated at the three public psychiatric institutions in the country, two of which are located in the Greater Accra region of Ghana. Owing to stigma, most people are not willing to visit these facilities for support. Although there are ongoing efforts to decentralize and integrate the provision of mental health services into primary healthcare [33], the limited publicity of these services means that several people, including pregnant women, may not be aware of their availability. The traditional world view of Ghanaians regarding demons or supernatural forces as causes of mental disorders places additional constraints on pregnant women's willingness to initiate mental health help-seeking from friends and family members and health professionals, despite the documented benefits of doing so [13].

The study showed that few pregnant women who reportedly sought help on their own for mental health problems were more likely to be women with obstetric history, were victims of intimate partner violence, had sleep difficulty, experienced suicidal ideation and reported low social support. This observation largely supports the existing literature on the relationship between exposure to risk factors and utilization of mental health services [27, 31, 32].

Health Professionals Involvement in Promoting Mental Health in Pregnancy

The observation that pregnant women infrequently seek mental health support on their own volition as reported in this and previous studies [11] calls for more pragmatic efforts from health professionals. In this study, more than half of the pregnant women reported that nurses/midwives asked about their emotional or mental well-being. This observation is largely consistent with an earlier study in Ghana in which more than half of the nurses and midwives reported that they were involved in promoting maternal mental health such as asking pregnant women about their emotional well-being [9]. Demographically, pregnant women in the second trimester and those with a high school diploma or equivalent were more likely to be asked about their mental and emotional well-being. Anecdotally, most pregnant women in Ghana begin attending antenatal clinics in their second trimester. This raises the possibility that a comprehensive intake assessment undertaken for first time antenatal clinic attendees may comprise an assessment of their mental and emotional well-being. The documented relationship between low education and risk for poor mental health [34] could partly explain why pregnant women with secondary school education in this study reported that health professionals enquired about their emotional and mental well-being.

When demographic variables were controlled for, sleep difficulty emerged as the robust predictor of the pregnant women being asked about mental and emotional well-being as well as

being offered mental health support such as counselling and referral services. Poor sleep not only negatively impacts mental health [35], but it is also a symptom of poor mental health (Anderson & Bradley, 2013). Therefore, sleep difficulty is likely to come to the attention of health professionals as a contributory factor to the mental health challenges reported by pregnant women. Pregnant women who feared vaginal delivery and expressed concerns about COVID-19 were reportedly offered mental health support. In a population-based cohort study, fear of vaginal delivery was strongly associated with a preference for elective caesarean section [36]. Estimated to range from 7%-27%, elective caesarean section is a major concern among researchers, policy-makers, stakeholders and health professionals owing to the associated surgical complications [37] and perhaps costs and consequences for future pregnancies. Efforts to persuade pregnant women capable of vaginal delivery to do so may involve offering them mental health support, including counselling and providing referral sources to allay fear and anxiety. A similar argument could be advanced for concerns regarding COVID-19. That is, health professionals may offer mental health support to reduce the fears and anxiety surrounding COVID-19 as a way of encouraging the continuous use of antenatal services and to promote delivery at health facilities in the COVID-19 pandemic.

Lastly, nurses and midwives enquired about the mental health or emotional wellbeing of more than half of the participants. In terms of support, about 67% of the participants received some mental health support. Perhaps, those who received support were those who indicated poor mental health or emotional distress to the nurses and midwives. There is also the possibility that some pregnant women may not be offered mental health support, although they may indicate problems with their mental health or emotional well-being. While the reasons accounting for this were not explored in this study, a previous study identified barriers hampering the involvement

of nurses and midwives in perinatal mental health in Ghana. These include lack of knowledge about mental health in pregnancy, unavailability of mental health services, and lack of a clear mental healthcare pathway [8].

Limitations

The study findings should be evaluated in light of the following limitations. The cross-sectional design adopted in this study does not permit commentaries on causal relationship. The recruitment of participants with at least senior high school education for the study implies that the findings may not be applicable to those with less formal education. Therefore, the application of the study findings should be restricted to pregnant women who have attended at least secondary education in Ghana. As noted previously, most pregnant women start antenatal clinics in their second trimester, making it difficult to obtain a representation of this category of women. The findings could have been different with equal or similar number of pregnant women across the three trimesters. The use of convenience sampling increases the risk of selection bias that may impact the study result negatively. Indeed, because the participants were self-selecting, there is a possibility that only those with unique characteristics (e.g., interest in research) agreed to participate in the study. This line of reasoning further limits the application of the study findings to a broader population of pregnant women with at least senior secondary education. The choice of the study settings does not include government owned health facilities and it is not clear whether similar findings would be obtained from government facilities. Lastly, although single or few-item measures are capable of representing complex variables, multiple-item questionnaires are known to have superior psychometric properties, which could have influenced the findings reported here [38, 39].

Conclusion

Pregnancy is a period of vulnerability for women to experience poor mental health. Several pregnancy-related stressors are unavoidable and many that are preventable continue to exist and manifest in diverse forms. Providing mental health support early enough to pregnant women could help to mitigate the impact of stressors on their mental and emotional well-being as well as the development of their children. While it is important to promote self-initiated mental health help-seeking, it is equally imperative to involve health professionals in the provision of mental health support, as advocated by the WHO’s task shifting concept [40]. Both individual and health professional-initiated activation of the mental health pathway will ultimately benefit pregnant women and their unborn babies. With evidence suggesting high mental health illiteracy among pregnant women [41], greater responsibility is placed on health professionals to intervene in providing mental health services to pregnant women, as advocated for by the NICE service management and clinical guidance for antenatal and postnatal mental health [16]. Health professionals should be well positioned to identify or detect pregnant women in need of mental health services and activate the pathway towards seeking help. The findings reported here re-emphasize the need for profiling pregnant women. To understand pregnant women who are likely to seek mental health support on their own, the study suggests that attention should be paid to those with the following background: History of hypertension or diabetes in the past and present pregnancies, victims of intimate partner violence, sleep difficulty, suicidal ideation and low social support. Health professionals can target pregnant women with the aforementioned experiences, in addition to those who feared vaginal delivery and expressed concern about COVID-19, to improve their mental and emotional well-being.

Acknowledgement

The project is an OPERA (Optimal Pregnancy Environment Risk Association) project. We acknowledge the Canadian Queen Elizabeth II Diamond Jubilee Scholarships-Advanced Scholars (QES-AS) program. The QES is managed through a unique partnership of Universities Canada, the Rideau Hall Foundation (RHF), Community Foundations of Canada (CFC) and Canadian universities. The QES-AS is made possible with financial support from IDRC and SSHRC."

Declaration of interests

The authors declare that they have no known competing financial interests or personal relationships that could have appeared to influence the work reported in this paper.

References

1. Grose RG, Roof KA, Semenza DC, Leroux X, Yount KM. Mental health, empowerment, and violence against young women in lower-income countries: A review of reviews. Aggression and violent behavior. 2019;46:25-36.

2. Cohen E, Ware LJ, Prioreschi A, Draper C, Bosire E, Lye SJ, et al. Material and Relational Difficulties: The Impact of the Household Environment on the Emotional Well-Being of Young Black Women Living in Soweto, South Africa. Journal of Family Issues. 2020;41(8):1307-32.

3. Sawyer A, Ayers S, Smith H. Pre-and postnatal psychological wellbeing in Africa: a systematic review. Journal of affective disorders. 2010;123(1-3):17-29.

4. Ghimire U, Papabathini SS, Kawuki J, Obore N, Musa TH. Depression during pregnancy and the risk of low birth weight, preterm birth and intrauterine growth restriction-an updated meta-analysis. Early Human Development. 2020;152:105243.

5. Rees S, Channon S, Waters CS. The impact of maternal prenatal and postnatal anxiety on children's emotional problems: a systematic review. European Child & Adolescent Psychiatry. 2019;28(2):257-80. doi: 10.1007/s00787-018-1173-5.

6. Slomian J, Honvo G, Emonts P, Reginster J-Y, Bruyère O. Consequences of maternal postpartum depression: A systematic review of maternal and infant outcomes. Women's Health. 2019;15:1745506519844044.

7. Weobong B, ten Asbroek AH, Soremekun S, Gram L, Amenga-Etego S, Danso S, et al. Association between probable postnatal depression and increased infant mortality and morbidity: findings from the DON population-based cohort study in rural Ghana. BMJ open. 2015;5(8).

8. Adjorlolo S, Aziato L. Barriers to addressing mental health issues in childbearing women in Ghana. Nursing Open. 2020;7(6):1779-86.

9. Adjorlolo S, Aziato L, Akorli VV. Promoting maternal mental health in Ghana: An examination of the involvement and professional development needs of nurses and midwives. Nurse education in practice. 2019;39:105-10.

10. Rickwood D, Deane FP, Wilson CJ, Ciarrochi J. Young people's help-seeking for mental health problems. Australian e-journal for the Advancement of Mental health. 2005;4(3):218-51.

11. Redshaw M, Henderson J. Who is actually asked about their mental health in pregnancy and the postnatal period? Findings from a national survey. BMC psychiatry. 2016;16(1):322.

12. Jesse DE, Dolbier CL, Blanchard A. Barriers to seeking help and treatment suggestions for prenatal depressive symptoms: Focus groups with rural low-income women. Issues in mental health nursing. 2008;29(1):3-19.

13. Barrera AZ, Nichols AD. Depression help-seeking attitudes and behaviors among an Internet-based sample of Spanish-speaking perinatal women. Revista panamericana de salud publica. 2015;37:148-53.

14. Jones A. Help seeking in the perinatal period: A review of barriers and facilitators. Social work in public health. 2019;34(7):596-605.

15. Badu E, Mitchell R, O'Brien AP. Pathways to mental health treatment in Ghana: Challenging biomedical methods from herbal-and faith-healing perspectives. International Journal of Social Psychiatry. 2019;65(6):527-38.

16. NICE. Antenatal and Postnatal Mental Health: Clinic Management and Service Guidance. London: 2014.

17. Reilly N, Harris S, Loxton D, Chojenta C, Forder P, Austin M-P. The impact of routine assessment of past or current mental health on help-seeking in the perinatal period. Women and Birth. 2014;27(4):e20-e7.

18. Smith MS, Lawrence V, Sadler E, Easter A. Barriers to accessing mental health services for women with perinatal mental illness: systematic review and meta-synthesis of qualitative studies in the UK. BMJ open. 2019;9(1):e024803.

19. Holt C, Milgrom J, Gemmill AW. Improving help-seeking for postnatal depression and anxiety: a cluster randomised controlled trial of motivational interviewing. Archives of women's mental health. 2017;20(6):791-801.

20. Da Costa D, Zelkowitz P, Nguyen T-V, Deville-Stoetzel J-B. Mental health help-seeking patterns and perceived barriers for care among nulliparous pregnant women. Archives of women's mental health. 2018;21(6):757-64.

21. Purtle J, Nelson KL, Counts NZ, Yudell M. Population-Based approaches to mental health: history, strategies, and evidence. Annual Review of Public Health. 2020;41:201-21.

22. Fuhrer R, Keyes KM. Population Mental Health in the 21st Century: Time to Act. American Public Health Association; 2019.

23. Whiteford HA, Degenhardt L, Rehm J, Baxter AJ, Ferrari AJ, Erskine HE, et al. Global burden of disease attributable to mental and substance use disorders: findings from the Global Burden of Disease Study 2010. The lancet. 2013;382(9904):1575-86.

24. Adjorlolo S, Abdul-Nasiru I, Chan HC, Bambi LE. Mental health professionals' attitudes toward offenders with mental illness (insanity acquittees) in Ghana. International journal of offender therapy and comparative criminology. 2018;62(3):629-54.

25. Ibrahim A, Hor S, Bahar OS, Dwomoh D, McKay MM, Esena RK, et al. Pathways to psychiatric care for mental disorders: a retrospective study of patients seeking mental health services at a public psychiatric facility in Ghana. International journal of mental health systems. 2016;10(1):1-11.

26. Molarius A, Berglund K, Eriksson C, Eriksson HG, Lindén-Boström M, Nordström E, et al. Mental health symptoms in relation to socio-economic conditions and lifestyle factors–a population-based study in Sweden. BMC public health. 2009;9(1):1-9.

27. Mandal SK, Hooker L, Vally H, Taft A. Partner violence and postnatal mental health: cross-sectional analysis of factors associated with depression and anxiety in new mothers. Australian journal of primary health. 2018;24(5):434-40.

28. Mertens G, Gerritsen L, Duijndam S, Salemink E, Engelhard IM. Fear of the coronavirus (COVID-19): Predictors in an online study conducted in March 2020. Journal of Anxiety Disorders. 2020;74:102258.

29. Kurtis MM, Balestrino R, Rodriguez-Blazquez C, Forjaz MJ, Martinez-Martin P. A review of scales to evaluate sleep disturbances in movement disorders. Frontiers in neurology. 2018;9:369.

30. Innamorati M, Pompili M, Serafini G, Lester D, Erbuto D, Amore M, et al. Psychometric properties of the suicidal history self-rating screening scale. Archives of Suicide Research. 2011;15(1):87-92.

31. Howard LM, Oram S, Galley H, Trevillion K, Feder G. Domestic violence and perinatal mental disorders: a systematic review and meta-analysis. PLoS Med. 2013;10(5):e1001452.

32. Leonardsson M, San Sebastian M. Prevalence and predictors of help-seeking for women exposed to spousal violence in India–a cross-sectional study. BMC women's health. 2017;17(1):99.

33. Akerele O. The decentralization of the ghanaian mental health system through the non-profit sector: a case to improve access to care and disrupt the “othering” of the mentally ill? Intersect: The Stanford Journal of Science, Technology, and Society. 2020;13(3).

34. Ten Kate J, De Koster W, Van der Waal J. Why are depressive symptoms more prevalent among the less educated? The relevance of low cultural capital and cultural entitlement. Sociological Spectrum. 2017;37(2):63-76.

35. Dixon CL. Sleep Deprivation & Pregnancy Related Risks. International Journal of Childbirth Education. 2014;29(3).

36. Størksen HT, Garthus-Niegel S, Adams SS, Vangen S, Eberhard-Gran M. Fear of childbirth and elective caesarean section: a population-based study. BMC pregnancy and childbirth. 2015;15(1):1-10.

37. Betrán AP, Ye J, Moller A-B, Zhang J, Gülmezoglu AM, Torloni MR. The increasing trend in caesarean section rates: global, regional and national estimates: 1990-2014. PloS one. 2016;11(2):e0148343.

38. Konstabel K, Lönnqvist J-E, Leikas S, García Velázquez R, Qin H, Verkasalo M, et al. Measuring single constructs by single items: Constructing an even shorter version of the “Short Five” personality inventory. PloS one. 2017;12(8):e0182714.

39. Cook CL, Perri III M. Single-item vs multiple-item measures of stage of change in compliance with prescribed medications. Psychological reports. 2004;94(1):115-24.

40. WHO. Task Shifting: Global Recommendations and Guidelines. Geneva: 2008.

41. Spedding MF, Stein DJ, Naledi T, Sorsdahl K. Pregnant women's mental health literacy and perceptions of perinatal mental disorders in the Western Cape, South Africa. Mental Health & Prevention. 2018;11:16-23.

Table 1: Prevalence of Self and Health Professional-Initiated Help-seeking for Mental Health among Pregnant Women (n = 702).

Variables/Response	Never	Yes	Once	Twice	Several times
	n (%)	*n* (%)	*n* (%)	*n* (%)	*n* (%)
**Self-Initiated Help-Seeking**					
Mental health seeking in last 3 months **(*n* = 687)**	557(81.1)	130(18.9)	83(12.1)	30(4.4)	17(2.5)
**Health Professional-Initiated Help-Seeking**					
Asked about mental health issues **(*n* = 685)**	241(35.2)	444(64.8)	227(33.1)	101(14.7)	116(16.9)
Offered mental health support **(*n*= 679)**	359(52.9)	320(47.1)	160(23.6)	78(11.5)	82(12.1)

Table 2: Frequencies and Chi Square Results for Demographic Correlates of the Outcome Variables.

Source	Self-Initiated Mental Health Help Seeking			Health Professional Initiated Mental Health Help Seeking			Health Professional Providing Mental Health Support		
	No *n* (%)	Yes *n* (%)	Value	No *n* (%)	Yes *n* (%)	Value	No *n* (%)	Yes *n* (%)	Value
**Age+**	-	-	.01, ns	-	-	.03, ns	-	-	.14**
**Research Setting**			96.07***			87.76***			208.11***
Legon	144 (21)	28(4.1)		75(10.9)	93(13.6)		122(18.0)	45(6.6)	
Alpha	210(37.7)	23(17.7)		122(17.8)	110(16.1)		174(25.6)	55(8.1)	
Sanford	37(5.4)	49(7.1)		18(2.6)	68(9.9)		38(5.6)	46(6.8)	
St-Gregory	166(24.2)	30(4.4)		26(3.8)	173(25.3)		25(3.7)	174(25.6)	
Total	557(81.1)	130(18.9)		241(35.2)	444(64.8)		359(52.9)	320(47.1)	
**Trimester**			.39, ns			23.96***			31.70***
First	52(7.6)	11(1.6)		25(3.6)	36(5.3)		36(5.3)	25(3.7)	
Second	245(35.7)	61(8.9)		77(11.2)	228(33.3)		124(18.3)	179(26.4)	
Third	260(35.8)	58(8.4)		139(20.3)	180(26.3)		199(29.3)	116(17.1)	
Total	557(81.1)	130(18.9)		241(35.2)	444(64.8)		359(52.9)	320(47.1)	
**Education**			4.40, ns			46.65***			48.80***
SHS/below	276(41.6)	72(10.9)		102(15.5)	249(37.7)		154(23.5)	194(29.7)	
Diploma or equivalence	133(20.1)	33(5)		43(6.5)	121(18.3)		79(12.1)	84(12.8)	
First degree & above	129(19.5)	19(2.9)		85(12.9)	60(9.1)		112(17.1)	31(4.7)	
Total	538(81.3)	124(18.7)		230(34.8)	430(65.2)		345(52.8)	309(47.2)	
**Marital Status**			16.37***			3.02, ns			1.84, ns
Single	86(12.6)	39(5.7)		35(5.2)	89(13.1)		58(8.6)	65(9.7)	
Married	469(68.9)	87(12.8)		202(29.8)	352(51.9)		296(44)	253(37.6)	
Total	555(81.5)	126(18.5)		237(35)	441(65)		354(52.7)	318(47.3)	
**Employment**			2.91, ns			.44, ns			8.22*
Formal	251(38.4)	69(10.6)		113(17.4)	201(30.9)		179(27.8)	134(20.8)	
Informal	157(24)	33(5.1)		65(10)	129(19.8)		85(13.2)	107(16.6)	
Unemployed	121(18.5)	22(3.4)		52(8)	90(13.8)		76(11.8)	63(9.8)	
Total	529(81)	124(19)		230(35.4)	420(64.6)		340(52.8)	304(47.2)	

* $p < .05$; ** $p < .01$, *** $p < .01$, ns = not significant.

Table 3: Chi Square and Point-Biserial Correlation Interrelations Results for Study Variables

	1	2	3	4	5	6	7	8	9	10	11	12
**Self-Initiated Help-Seeking**												
**1.** Last 3 month	1											
**Professional Initiated Help-Seeking**												
**2.** Asked	27.72**	1										
**3.** Support offered	18.31**	209.11**	1									
**Medical History**												
**4.** Past	34.48**	5.39*	14.16**	1								
**5.** Present	27.97**	10.07**	13.26**	320.79**	1							
**Fear of Delivery**												
**6.** Vaginal delivery	27.08**	16.98**	50.28**	1.03	.63	1						
**7.** Cesarean Section	2.01	8.60**	26.04**	2.76	.01	28.24**	1					
**Intimate Partner Violence**												
**8.** Partner Neglect	11.24**	.46	.51	11.34**	13.40**	2.48	.30	1				
**9.** Partner abuse	26.32**	7.67**	7.67**	11.30**	15.13**	3.90*	17.39	.40	1			
**10. Social Support+**	-.36**	-.08*	-.03	.14**	.21**	.14**	.05	.25**	-.31**	1		
**11. COVID-19+ concern**	.09*	.26***	.41**	-.21***	-.13**	-.26**	-.33**	-.04	.17**	.06	1	
**12. Sleep Difficulty+**	.21**	.22***	.31**	-.10**	-.12**	-.30**	-.24**	-.11**	.14**	-.08*	.47**	1
**13. Suicidal Ideation+**	.31**	.11**	.08*	-.09*	-.12**	-.17**	-.07	-.21**	.16**	-.39**	.05	.17**

* p < .05; ** p < .01; + = point-biserial correlation coefficients.

Table 4: Predictors of Mental Health Help-Seeking

	Self- Help-seeking		Asked about Mental health		Offered Mental Health Support	
	OR (95% CI)	AOR (95% CI)	OR (95% CI)	AOR (95% CI)	OR (95% CI)	AOR (95% CI)
**Medical history**						
Past	3.90***(2.419-6.289)	3.58***(2.071-6.184)	1.83*(1.093-3.079)	1.18(0.639-2.158)	2.42***(1.511-3.870)	1.21(0.608-2.412)
Present	3.13**(1.970-4.979)	2.26**(1.328-3.847)	2.25**(1.349-3.74)	1.67(0.922-3.021)	2.24***(1.439-3.474)	1.40(0.736-2.669)
**Fear of delivery**						
Vaginal delivery	2.91***(1.926-4.395)	1.94**(1.186-3.185)	2.17***(1.496-3.157)	1.13(0.714-1.788)	3.47***(2.444-4.938)	2.13**(1.254-3.603)
C- section	1.38(0.883-2.156)	1.29(0.770-2.153)	1.68**(1.185-2.371)	1.01(0.672-1.504)	2.45***(1.728-3.463)	1.17(0.721-1.912)
**Partner violence**						
Partner neglect	.44**(0.274-720)	.47*(0.266-838)	1.17(0.746-1.829)	1.38(0.807-2.351)	.85(0.553-1.319)	.87(0.461-1.655)
Partner abuse	2.73***(1.845-4.045)	2.12*(1.362-3.301)	1.61**(1.148-2.260)	1.12(0.753-1.667)	1.56**(1.139-2.145)	1.11(0.697-1.750)
**Social support**	.70***(0.648-.762)	.77***(0.695-845)	.93*(0.869-.996)	.95(0.867-1.034)	.98(0.920-1.043)	.97(0.869-1.071)
**COVID-19**	1.10*(1.014-1.202)	1.15*(1.017-1.035)	1.30***(1.205-1.411)	1.09(.987-1.211)	1.51***(1.391-1.631)	1.21**(1.082-1.359)
**Sleep difficulty**	1.38***(1.227-1.548)	1.37***(1.191-1.586)	1.36***(1.221-1.515)	1.15*(1.011-1.305)	1.52***(1.364-1.686)	1.18*(1.020-1.363)
**Suicidal ideation**	1.61***(1.415-1.839)	1.40***(1.208-1.619)	1.21(1.056-1.378)	1.16(0.997-1.349)	1.13*(1.006-1.261)	1.12(0.954-1.303)

* $p < 0.05$; ** $p < 0.01$, *** $p < 0.001$; C –section = Caesarean section

Adjusted odds ratios were obtained after controlling for demographic variables that evinced significant effect on the outcome variables (Table 2).

Table 5: Predictors of Mental Health Help-Seeking, Controlling for Individual Predictors and Demographic Variables.

	Help-seeking last 1 month	Asked about Mental health	Offered Mental Health Support
	AOR (95% CI)	AOR (95% CI)	AOR (95% CI)
**Medical history**			
Past	3.80** (1.636-8.842)	.74(.320-1.706)	.82(.317-2.127)
Present	0.75 (0.326-1.747)	1.97(.858-4.509)	1.24(.496-3.088)
**Fear of delivery**			
Vaginal delivery	1.59(.903-2.792)	1.07(.662-1.743)	1.91*(1.097-3.312)
Caesarean section	.78(.422-1.433)	.82(.526-1.275)	1.06(.630-1.768)
**Partner violence**			
Partner neglect	.74(.367-1.471)	1.46(.775-2.757)	.85(.392-1.823)
Partner abuse	1.88*(1.091-3.288)	1.11(.703-1.750)	1.11(.655-1.882)
**Social support**	.80***(.711-908)	.96(.863-1.063)	.95(.842-1.080)
**COVID-19 worries**	1.07(.905-1.261)	1.09(.971-1.234)	1.19*(1.038-1.354)
**Sleep difficulty**	1.35**(1.125-1.626)	1.04(.896-1.201)	1.05(.881-1.239)
**Suicidal ideation**	1.22**(1.092-1.449)	1.13(.950-1.339)	1.09(.916-1.307)

* $p < .05$; ** $p < .01$, *** $p < .001$

Demographic variables that evinced significant effect on the outcome variables (Table 2) were controlled for.
