# Supplementary material for: Seeking and receiving help for mental health services among pregnant women in Ghana
Source: PLoS One. 2023 Mar 3;18(3):e0280496. doi: 10.1371/journal.pone.0280496 (PMC9983869; doi:10.1371/journal.pone.0280496)
Supplement: Attachment — Submitted filename: second review of mental health among pregnant women - Copy.docx [file pone.0280496.s002.docx]

The authors have reworked on the write-up on “Seeking and Receiving Help for Mental Health Services Among Pregnant Women in Ghana” based on the review suggestions made. The manuscript makes interesting reading and has the potential of contributing significantly towards the care given to pregnant women.

On page 9, authors indicated the type of sampling used using the sentence “Participants were recruited

on based on convenience.” It is suggested that they use “Convenient sampling was used to recruit study participants.”
